# No-Dimensional Tverberg Theorems and Algorithms

**DOI:** 10.1007/s00454-022-00380-1

**Published:** 2022-04-12

**Authors:** Aruni Choudhary, Wolfgang Mulzer

**Affiliations:** Institut für Informatik, Freie Universität Berlin, Berlin, Germany

**Keywords:** Tverberg theorem, Colorful Carathéodory theorem, Approximation algorithm, 68W25, 52C99

## Abstract

Tverberg’s theorem states that for any $k\ge 2$ and any set $P\subset {\mathbb {R}}^d$ of at least $(d+1)(k-1)+1$ points in *d* dimensions, we can partition *P* into *k* subsets whose convex hulls have a non-empty intersection. The associated search problem of finding the partition lies in the complexity class $\text {CLS} = \text {PPAD} \cap \text {PLS}$, but no hardness results are known. In the *colorful* Tverberg theorem, the points in *P* have colors, and under certain conditions, *P* can be partitioned into *colorful* sets, in which each color appears exactly once and whose convex hulls intersect. To date, the complexity of the associated search problem is unresolved. Recently, Adiprasito, Bárány, and Mustafa (SODA 2019) gave a *no-dimensional* Tverberg theorem, in which the convex hulls may intersect in an *approximate* fashion. This relaxes the requirement on the cardinality of *P*. The argument is constructive, but does not result in a polynomial-time algorithm. We present a deterministic algorithm that finds for any *n*-point set $P\subset {\mathbb {R}}^d$ and any $k\in \{2,\dots ,n\}$ in $O(nd\lceil {\log k}\rceil )$ time a *k*-partition of *P* such that there is a ball of radius $O((k/\sqrt{n}){\text {diam}}(P))$ that intersects the convex hull of each set. Given that this problem is not known to be solvable exactly in polynomial time, our result provides a remarkably efficient and simple new notion of approximation. Our main contribution is to generalize Sarkaria’s method (Israel Journal Math., 1992) to reduce the Tverberg problem to the colorful Carathéodory problem (in the simplified tensor product interpretation of Bárány and Onn) and to apply it algorithmically. It turns out that this not only leads to an alternative algorithmic proof of a no-dimensional Tverberg theorem, but it also generalizes to other settings such as the colorful variant of the problem.

## Introduction

In 1921, Radon [27] proved a seminal theorem in convex geometry: given a set *P* of at least $d + 2$ points in ${\mathbb {R}}^d$, one can always split *P* into two non-empty sets whose convex hulls intersect. In 1966, Tverberg [34] generalized Radon’s theorem to allow for more sets in the partition. Specifically, he showed that for any $k \ge 1$, if a *d*-dimensional point set $P\subset {\mathbb {R}}^d$ has cardinality at least $(d + 1)(k - 1) + 1$, then *P* can be partitioned into *k* non-empty, pairwise disjoint sets $T_1, \dots , T_k \subset P$ whose convex hulls have a non-empty intersection, i.e., $\bigcap _{i = 1}^k \mathrm {conv}(T_i) \ne \emptyset$, where $\mathrm {conv}(\,{\cdot }\,)$ denotes the convex hull.

**Fig. 1:**
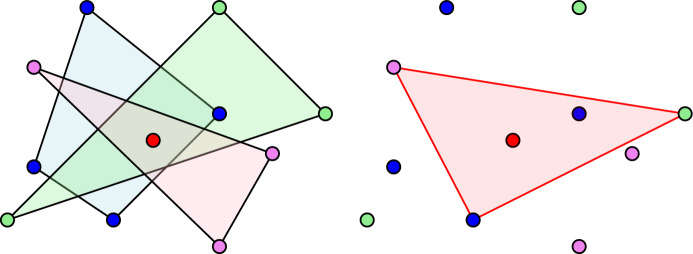
The colorful Carathéodory theorem. Left: the convex hulls of the three point sets intersect; Right: a colorful triangle that contains the common point

By now, several alternative proofs of Tverberg’s theorem are known, e.g., [3, 5, 8, 21, 28, 29, 35, 36]. Perhaps the most elegant proof is due to Sarkaria [29], with simplifications by Bárány and Onn [8] and by Aroch et al. [3]. In this paper, all further references to *Sarkaria’s method* refer to the simplified version. This proof proceeds by a reduction to the *colorful Carathéodory theorem*, another celebrated result in convex geometry: given $r \ge d + 1$ point sets $P_1, \dots , P_r \subset {\mathbb {R}}^d$ that have a common point *y* in their convex hulls $\mathrm {conv}(P_1), \dots , \mathrm {conv}(P_r)$, there is a *traversal* $x_1 \in P_1, \dots , x_r \in P_r$, such that $\mathrm {conv}(\{x_1, \dots , x_r\})$ contains *y*. A two-dimensional example is given in Fig. 1. Sarkaria’s proof [29] uses a tensor product to lift the original points of the Tverberg instance into higher dimensions, and then uses the colorful Carathéodory traversal to obtain a Tverberg partition for the original point set.

From a computational point of view, a Radon partition is easy to find by solving $d + 1$ linear equations. On the other hand, finding Tverberg partitions is not straightforward. Since a Tverberg partition must exist if *P* is large enough, finding such a partition is a total search problem. In fact, the problem of computing a colorful Carathéodory traversal lies in the complexity class $CLS = PPAD \cap \text {PLS}$ [20, 23], but no better upper bound is known. Sarkaria’s proof gives a polynomial-time reduction from the problem of finding a Tverberg partition to the problem of finding a colorful traversal, thereby placing the former problem in the same complexity class. Again, as of now we do not know better upper bounds for the general problem. Miller and Sheehy [21] and Mulzer and Werner [24] provided algorithms for finding *approximate* Tverberg partitions, computing a partition into fewer sets than is guaranteed by Tverberg’s theorem in time that is linear in *n*, but quasi-polynomial in the dimension. These algorithms were motivated by applications in mesh generation and statistics that require finding a point that lies “deep” in *P*. A point in the common intersection of the convex hulls of a Tverberg partition has this property, with the partition serving as a certificate of depth. Recently Har-Peled and Zhou have proposed algorithms [16] to compute approximate Tverberg partitions that take time polynomial in *n* and *d*.

Tverberg’s theorem also admits a colorful variant, first conjectured by Bárány and Larman [7]. The setup consists of $$d + 1$$ point sets $$P_1, \dots , P_{d+1} \subset {\mathbb {R}}^d$$, each set interpreted as a different color and having size *t*. For a given *k*, the goal is to find *k* pairwise-disjoint *colorful* sets (i.e., each set contains at most one point from each $$P_i$$) $$A_1,\dots ,A_k$$ such that $$\bigcap _{i=1}^{k} \mathrm {conv}(A_i) \ne \emptyset $$. The problem is to determine the optimal value of *t* for which such a colorful partition always exists. Bárány and Larman [7] conjectured that $$t=k$$ suffices and they proved the conjecture for $$d = 2$$ and arbitrary *k*, and for $$k =2 $$ and arbitrary *d*. The first result for the general case was given by Živaljević and Vrećica [38] through topological arguments. Using another topological argument, Blagojević et al. [9] showed that (i) if $$k + 1$$ is prime, then $$t = k$$; and (ii) if $$k + 1$$ is not prime, then $$k\le t \le 2k-2$$. These are the best known bounds for arbitrary *k*. Later Matoušek et al. [19] gave a geometric proof that is inspired by the proof of Blagojević et al. [9].

More recently, Soberón [30] showed that if more color classes are available, then the conjecture holds for any *k*. More precisely, for $P_1,\dots ,P_n \subset {\mathbb {R}}^d$ with $n = (k - 1)d + 1$, each of size *k*, there exist *k* colorful sets whose convex hulls intersect. Moreover, there is a point in the common intersection so that the coefficients of its convex combination are the same for each colorful set in the partition. The proof uses Sarkaria’s tensor product construction.

Recently Adiprasito et al. [1] established a relaxed version of the colorful Carathéodory theorem and some of its descendants [4]. For the colorful Carathéodory theorem, this allows for a (relaxed) traversal of arbitrary size, with a guarantee that the convex hull of the traversal is close to the common point *y*. For the colorful Tverberg problem, they prove a version of the conjecture where the convex hulls of the colorful sets intersect approximately. This also gives a relaxation for Tverberg’s theorem [34] that allows arbitrary-sized partitions, again with an approximate notion of intersection. Adiprasito et al. refer to these results as *no-dimensional* versions of the respective classic theorems, because the dependence on the ambient dimension is relaxed. The proofs use averaging arguments. The argument for the no-dimensional colorful Carathéodory theorem also gives an efficient algorithm to find a suitable traversal. However, the arguments for the no-dimensional Tverberg theorem results do not give a polynomial-time algorithm for finding the partitions.

*Our contributions.* We prove no-dimensional variants of the Tverberg theorem and its colorful counterpart that allow for efficient algorithms. Our proofs are inspired by Sarkaria’s method [29] and the averaging technique by Adiprasito, Bárány, and Mustafa [1]. For the colorful version, we additionally make use of ideas of Soberón [30]. Furthermore, we also give a no-dimensional generalized Ham-Sandwich theorem [37] that interpolates between the Centerpoint Theorem and the Ham-Sandwich Theorem [33], again with an efficient algorithm.

Algorithmically, Tverberg’s theorem is useful for finding centerpoints of high-dimensional point sets, which in turn has applications in statistics and mesh generation [21]. In fact, most algorithms for finding centerpoints are Monte-Carlo, returning some point *p* and a probabilistic guarantee that *p* is indeed a centerpoint [11, 15]. However, this is coNP-hard to verify. On the other hand, a (possibly approximate) Tverberg partition immediately gives a certificate of depth [21, 24]. Unfortunately, there are no polynomial-time algorithms for finding optimal Tverberg partitions. In this context, our result provides a fresh notion of approximation that also leads to very fast polynomial-time algorithms.

Furthermore, the Tverberg problem is intriguing from a complexity theoretic point of view, because it constitutes a total search problem that is not known to be solvable in polynomial time, but which is also unlikely to be NP-hard. So far, such problems have mostly been studied in the context of algorithmic game theory [25], and only very recently a similar line of investigation has been launched for problems in high-dimensional discrete geometry [12, 14, 20, 23]. Thus, we show that the *no-dimensional* variant of Tverberg’s theorem is easy from this point of view. Our main results are as follows:

- Sarkaria’s method uses a specific set of *k* vectors in ${\mathbb {R}}^{k-1}$ to lift the points in the Tverberg instance to a colorful Carathéodory instance. We refine this method to vectors that are defined with the help of a given graph. The choice of this graph is important in proving good bounds for the partition and in the algorithm. We believe that this generalization is of independent interest and may prove useful in other scenarios that rely on the tensor product construction.
- Let ${\text {diam}}(x)$ denote the diameter of a set *x*. We prove an efficient no-dimensional Tverberg result:

### Theorem 1.1

(efficient no-dimensional Tverberg)   Let *P* be a set of *n* points in *d* dimensions, and let $k \in \{2, \dots , n\}$ be an integer.

(i) For any choice of positive integers $r_1,\dots ,r_k$ that satisfy $\sum _{i=1}^{k} r_i = n$, there is a partition $T_1, \dots , T_k$ of *P* with $|T_1| = r_1, |T_2|=r_2, \dots , |T_k|=r_k$, and a ball *B* of radius
  $$\begin{aligned} \frac{n{\text {diam}}(P)}{\min _i r_i} \sqrt{\frac{10 \lceil {\log _4 k}\rceil }{n-1}}=O\biggl (\frac{\sqrt{n\log k}}{\min _i r_i}{\text {diam}}(P)\biggr ) \end{aligned}$$
  such that *B* intersects the convex hull of each $T_i$.
(ii) The bound is better for the case $n=rk$ and $r_1=\ldots =r_k=r$. There exists a partition $T_1, \dots , T_k$ of *P* with $|T_1| = \ldots = |T_k| = r$ and a *d*-dimensional ball of radius
  $$\begin{aligned} \sqrt{\frac{k(k-1)}{n-1}}{\text {diam}}(P)=O\biggl (\frac{k}{\sqrt{n}}{\text {diam}}(P)\biggr )\end{aligned}$$
  that intersects the convex hull of each $T_i$.
(iii) In either case, the partition $T_1, \dots , T_k$ can be computed in deterministic time
  $$\begin{aligned} O(nd\lceil {\log k}\rceil ). \end{aligned}$$

**Fig. 2:**
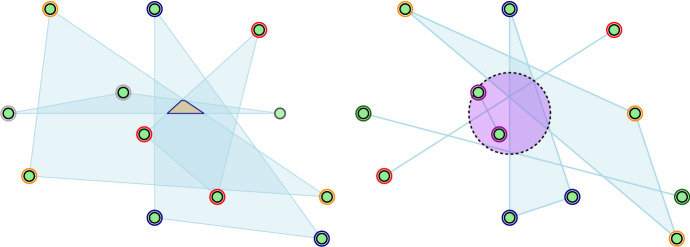
Left: a 4-partition of a planar point set. Larger Tverberg partitions are not possible because there are not enough points. Right: a 5-partition on the same point set with a disk intersecting the convex hulls of each set of the partition

See Fig. 2 for a simple illustration.

**Fig. 3:**
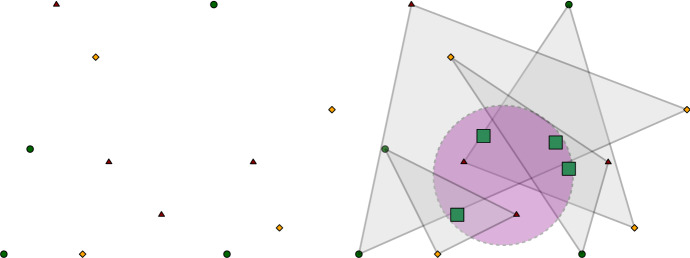
Left: a point set on three colors and four points of each color. Right: a colorful partition with a ball containing the centroids (squares) of the sets of the partition

- and a colorful counterpart (for a simple example, see Fig. 3):

### Theorem 1.2

(efficient no-dimensional colorful Tverberg)   Let $P_1$, $\ldots$, $P_n\,{\subset }\,\,{\mathbb {R}}^d$ be point sets, each of size *k*, with *k* being a positive integer, so that the total number of points is $N = nk$.

(i) Then, there are *k* pairwise-disjoint colorful sets $A_1,\dots ,A_k$ and a ball of radius
  $$\begin{aligned} \sqrt{\frac{2k(k-1)}{N}}\max _i{\text {diam}}(P_i)=O\biggl (\frac{k}{\sqrt{N}}\max _i{\text {diam}}(P_i)\biggr ) \end{aligned}$$
  that intersects $\mathrm {conv}(A_i)$ for each $i\in [k]$.
(ii) The colorful sets $A_1,\dots ,A_k$ can be computed in deterministic time *O*(*Ndk*).

- For any sets $P, x\subset {\mathbb {R}}^d$, the *depth* of *x* with respect to *P* is the largest positive integer *k* such that every half-space that contains *x* also contains at least *k* points of *P*.

### Theorem 1.3

(no-dimensional generalized Ham-Sandwich)   Let *k* finite point sets $P_1$, $\ldots$, $P_k$ in ${\mathbb {R}}^d$ be given, and let $m_1, \dots , m_k$, $2 \le m_i \le |P_i|$ for $i \in [k]$, $k\le d$, be any set of integers.

(i) There is a linear transformation and a ball $B\in {\mathbb {R}}^{d-k+1}$ of radius
  $$\begin{aligned}(2 + 2\sqrt{2}) \max _i \frac{{\text {diam}}(P_i)}{\sqrt{m_i}},\end{aligned}$$
  such that the hypercylinder $B\times {\mathbb {R}}^{k-1}\subset {\mathbb {R}}^d$ has depth at least $\lceil |P_i|/m_i\rceil$ with respect to $P_i$, for $i \in [k]$, after applying the transformation.
(ii) The ball and the transformation can be determined in time
  $$\begin{aligned}O\left( d^6+dk^2+\sum _{i} |P_i| d\right) .\end{aligned}$$

The colorful Tverberg result is similar in spirit to the regular version, but from a computational viewpoint, it does not make sense to use the colorful algorithm to solve the regular Tverberg problem.

Compared to the results of Adiprasito et al. [1], our radius bounds are slightly worse. More precisely, they show that both in the colorful and the non-colorful case, there is a ball of radius $O\bigl (\sqrt{k/n}\,{\text {diam}}(P)\bigr )$ that intersects the convex hulls of the sets of the partition. They also show this bound is close to optimal. In contrast, our result is off by a factor of $O(\sqrt{k})$, but derandomizing the proof of Adiprasito et al. [1] gives only a brute-force $2^{O(n)}$-time algorithm. In contrast, our approach gives almost linear time algorithms for both cases, with a linear dependence on the dimension.

*Techniques.* Adiprasito et al. first prove the colorful no-dimensional Tverberg theorem using an averaging argument over an exponential number of possible partitions. Then, they specialize their result for the non-colorful case, obtaining a bound that is asymptotically optimal. Unfortunately, it is not clear how to derandomize the averaging argument efficiently. The method of conditional expectations applied to their averaging argument leads to a running time of $2^{O(n)}$. To get around this, we follow an alternate approach towards both versions of the Tverberg theorem. Instead of a direct averaging argument, we use a reduction to the colorful Carathéodory theorem that is inspired by Sarkaria’s proof, with some additional twists. We will see that this reduction also works in the no-dimensional setting, i.e., by a reduction to the no-dimensional colorful Carathéodory theorem of Adiprasito et al., we obtain a no-dimensional Tverberg theorem, with slightly weaker radius bounds, as stated above. This approach has the advantage that their colorful Carathéodory theorem is based on an averaging argument that permits an efficient derandomization using the method of conditional expectations [2]. In fact, we will see that the special structure of the no-dimensional colorful Carathéodory instance that we create allows for a very fast evaluation of the conditional expectations, as we fix the next part of the solution. This results in an algorithm whose running time is $O(n d \lceil {\log k}\rceil )$ instead of *O*(*ndk*), as given by a naive application of the method. With a few interesting modifications, this idea also works in the colorful setting. This seems to be the first instance of using Sarkaria’s method with special lifting vectors, and we hope that this will prove useful for further studies on Tverberg’s theorem and related problems.

*Updates from the conference version.* An extended abstract [10] of this work appeared at the 36th International Symposium on Computational Geometry. The conference abstract omitted the details of the results of Theorems 1.2 and 1.3. In this version, we present all the missing details.

*Outline of the paper.* We describe our extension of Sarkaria’s technique in Sect. 2 and an averaging argument that is essential for our results. In Sect. 3, we present the proof of the no-dimensional Tverberg theorem (Theorem 1.1). The algorithm for computing the partition is also detailed therein. Section 4 contains the results for the colorful setting of Tverberg (Theorem 1.2) and Sect. 5 presents results for the generalized Ham-Sandwich theorem (Theorem 1.3). We conclude in Sect. 6 with some observations and open questions.

## Tensor Product and Averaging Argument

Let $P \subset {\mathbb {R}}^d$ be the given set of *n* points. We assume for simplicity that the centroid of *P*, that we denote by *c*(*P*), coincides with the origin ${\mathbf {0}}$, that is, $\sum _{x \in P} x = {\mathbf {0}}$. For ease of presentation, we denote the origin by ${\mathbf {0}}$ in all dimensions, as long as there is no danger of ambiguity. Also, we write $\langle \,{\cdot }\,,\,{\cdot }\,\rangle$ for the usual scalar product between two vectors in the appropriate dimension, and [*n*] for the set $\{1, \dots , n\}$.

### Tensor Product

Let $x = (x_1, \dots , x_d) \in {\mathbb {R}}^d$ and $y = (y_1, \dots , y_m) \in {\mathbb {R}}^m$ be any two vectors. The *tensor product* $\otimes$ is the operation that takes *x* and *y* to the *dm*-dimensional vector $x\otimes y$ whose *ij*-th component is $x_i y_j$, that is,

$$\begin{aligned} x\otimes y= (xy_1, \dots , xy_m) =(x_1y_1, \dots ,x_dy_1, x_1y_2,\dots , x_d y_{m-1}, \dots ,x_dy_m) \in {\mathbb {R}}^{dm}.\end{aligned}$$

Easy calculations show that for any $x, x'\in {\mathbb {R}}^d, y, y'\in {\mathbb {R}}^m$, the operator $\otimes$ satisfies:

(i) $$x\otimes y + x'\otimes y = (x+x')\otimes y$$;
(ii) $$x\otimes y + x\otimes y' = x\otimes (y+y')$$; and
(iii) $$\langle x\otimes y,x'\otimes y'\rangle = \langle x,x'\rangle \langle y,y'\rangle $$.

By (iii), the $L_2$-norm $\Vert x\otimes y \Vert$ of the tensor product $x\otimes y$ is exactly $\Vert x\Vert \Vert y\Vert$. For any set of vectors $X = \{x_1, x_2, \ldots \}$ in ${\mathbb {R}}^d$ and any *m*-dimensional vector $q \in {\mathbb {R}}^m$, we denote by $X \otimes q$ the set of tensor products $\{x_1 \otimes q, x_2 \otimes q, \dots \} \subset {\mathbb {R}}^{dm}$. Throughout this paper, all distances will be measured in the $L_2$-norm.

*A set of lifting vectors.* We generalize the tensor construction that was used by Sarkaria to prove the Tverberg theorem [29]. For this, we provide a way to construct a set of *k* vectors $\{q_1, \dots , q_k\}$ that we use to create tensor products. The motivation behind the precise choice of these vectors will be clear in the next section, when we apply the construction to prove the no-dimensional Tverberg result. Let ${\mathcal {G}}$ be an (undirected) simple, connected graph of *k* nodes. Let

- $\Vert {\mathcal {G}}\Vert$ denote the number of edges in ${\mathcal {G}}$,
- $\varDelta ({\mathcal {G}})$ denote the maximum degree of any node in ${\mathcal {G}}$, and
- ${\text {diam}}({\mathcal {G}})$ denote the diameter of ${\mathcal {G}}$, i.e., the maximum length of a shortest path between a pair of vertices in ${\mathcal {G}}$.

We orient the edges of ${\mathcal {G}}$ in an arbitrary manner to obtain an oriented graph. We use this directed version of ${\mathcal {G}}$ to define a set of *k* vectors $\{q_1, \dots , q_k\}$ in $\Vert {\mathcal {G}}\Vert$ dimensions. This is done as follows: each vector $q_i$ corresponds to a unique node $v_i$ of ${\mathcal {G}}$ and its co-ordinates correspond to the row in the oriented incidence matrix assigned to $v_i$. More precisely, each coordinate position of the vectors corresponds to a unique edge of ${\mathcal {G}}$. If $v_iv_j$ is a directed edge of ${\mathcal {G}}$, then $q_i$ contains a 1 and $q_j$ contains a $-1$ in the corresponding coordinate position. The remaining co-ordinates are zero. That means, the vectors $\{q_1, \dots , q_k\}$ are in ${\mathbb {R}}^{\Vert {\mathcal {G}}\Vert }$. Also, $\sum _{i = 1}^{k} q_i = {\mathbf {0}}$. It can be verified that this is the unique linear dependence (up to scaling) between the vectors for any choice of edge orientations of ${\mathcal {G}}$. This means that the rank of the matrix with the $q_i$’s as the rows is $k-1$. It can be verified that:

#### Lemma 2.1

For each vertex $v_i$, the squared norm $\Vert q_i\Vert ^2$ is the degree of $v_i$. For $i\ne j$, the dot product $\langle q_i,q_j\rangle$ is $-1$ if $v_iv_j$ is an edge in ${\mathcal {G}}$, and 0 otherwise.

An immediate application of Lemma 2.1 and property (iii) of the tensor product is that for any set of *k* vectors $\{u_1,\dots ,u_k\}$, each of the same dimension, the following relation holds:

1 $$\begin{aligned} \begin{aligned} \left\| \sum _{i=1}^{k} u_i\otimes q_i \right\| ^2&=\,\sum _{i=1}^{k} \sum _{j=1}^{k} \langle u_i\otimes q_i,u_j\otimes q_j\rangle = \sum _{i=1}^{k} \sum _{j=1}^{k} \langle u_i,u_j\rangle \langle q_i,q_j\rangle \\&= \,\sum _{i=1}^{k} \langle u_i,u_i\rangle \langle q_i,q_i\rangle +2\sum _{1\le i<j\le k}^{k} \langle u_i,u_j\rangle \langle q_i,q_j\rangle \\&=\,\sum _{i=1}^{k} \Vert u_i\Vert ^2 \Vert q_i\Vert ^2-2\sum _{v_iv_j\in E} \langle u_i,u_j\rangle =\sum _{v_iv_j\in E} \Vert u_i-u_j\Vert ^2, \end{aligned} \end{aligned}$$

where *E* is the set of edges of ${\mathcal {G}}$.^1^

One of the simplest examples of such a set can be formed by selecting ${\mathcal {G}}$ to be the star graph. Each of the $k-1$ leaves correspond to a standard basis vector of ${\mathbb {R}}^{k-1}$ and the root corresponds to $(-1,\dots ,-1)\in {\mathbb {R}}^{k-1}$. This is also the set used in Bárány and Onn’s interpretation [8] of Sarkaria’s proof.

A more sophisticated example can be formed by taking ${\mathcal {G}}$ as a balanced binary tree with *k* nodes, and orienting the edges away from the root. Let $q_1$ correspond to the root. A simple instance of the vectors is shown below: 
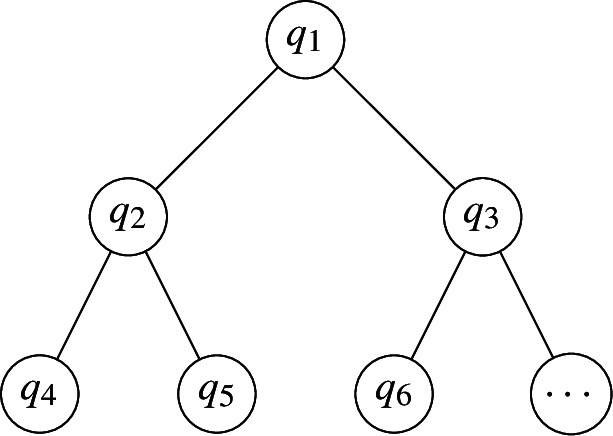
 The vectors in the figure above can be represented as the matrix

$$\begin{aligned} \begin{pmatrix} q_1\\ q_2\\ q_3\\ q_4\\ q_5\\ q_6\\ \dots \\ \end{pmatrix}=\begin{pmatrix} 1 &{} 1 &{} 0 &{} 0 &{} 0 &{} 0 &{} 0 &{} 0 &{}\dots \\ -1 &{} 0 &{} 1 &{} 1 &{} 0 &{} 0 &{} 0 &{} 0 &{}\dots \\ 0 &{}-1 &{} 0 &{} 0 &{} 1 &{} 1 &{} 0 &{} 0 &{}\dots \\ 0 &{} 0 &{}-1 &{} 0 &{} 0 &{} 0 &{} 1 &{} 1 &{}\dots \\ 0 &{} 0 &{} 0 &{}-1 &{} 0 &{} 0 &{} 0 &{} 0 &{} \dots \\ 0 &{} 0 &{} 0 &{} 0 &{} -1 &{} 0 &{} 0 &{} 0 &{} \dots \\ &{}&{}&{}\dots &{}&{}&{} \end{pmatrix} \end{aligned}$$

where the *i*-th row of the matrix corresponds to vector $q_i$. As $\Vert {\mathcal {G}}\Vert =k-1$, each vector is in ${\mathbb {R}}^{k-1}$. The norm $\Vert q_i\Vert$ is either $\sqrt{2}$, $\sqrt{3}$, or 1, depending on whether $v_i$ is the root, an internal node with two children, or a leaf, respectively. The height of ${\mathcal {G}}$ is $\lceil {\log k}\rceil$ and the maximum degree is $\varDelta ({\mathcal {G}})=3$.

### Averaging Argument

*Lifting the point set.* Let $P = \{p_1, \dots , p_n\}\subset {\mathbb {R}}^d$. We first pick a graph ${\mathcal {G}}$ with *k* vertices, as in the previous paragraph, and we derive a set of *k* lifting vectors $\{q_1, \dots , q_k\}$ from ${\mathcal {G}}$. Then, we lift each point of *P* to a set of vectors in $d\Vert {\mathcal {G}}\Vert$ dimensions, by taking tensor products with the vectors $\{q_1, \dots , q_k\}$. More precisely, for $a \in [n]$ and $j\in [k]$, let $p_{a,j} = p_a \otimes q_j \in {\mathbb {R}}^{d\Vert {\mathcal {G}}\Vert }$. For $a \in [n]$, we let $P_a = \{p_{a,1}, \dots ,p_{a,k}\}$ be the lifted points obtained from $p_a$. We have $\Vert p_{a,j}\Vert = \Vert q_j\Vert \Vert p_a\Vert \le \sqrt{\varDelta ({\mathcal {G}})}\Vert p_a\Vert$. By the bi-linear properties of the tensor product,

$$\begin{aligned} c(P_a) = \frac{1}{k} \sum _{j = 1}^{k}\,(p_a\otimes q_j)= \frac{1}{k}\left( p_a \otimes \left( \,\sum _{j=1}^{k} q_j \right) \right) = \frac{1}{k} ( p_a \otimes {\mathbf {0}})= {\mathbf {0}}, \end{aligned}$$

so the centroid $c(P_a)$ coincides with the origin, for $a \in [n]$.

The next lemma contains the technical core of our argument. The result is applied in Sect. 3 to derive a useful partition of *P* into *k* subsets of prescribed sizes from the lifted point sets.

#### Lemma 2.2

Let $P = \{p_1, \dots , p_n \}$ be a set of *n* points in ${\mathbb {R}}^d$ satisfying $\sum _{i=1}^{n} p_i = {\mathbf {0}}$. Let $P_1, \dots , P_n$ denote the point sets obtained by lifting each $p_i \in P$ using the vectors $\{ q_1, \dots , q_k \}$ defined using a graph ${\mathcal {G}}$.

(i) For any choice of positive integers $r_1,\dots ,r_k$ that satisfy $\sum _{i=1}^{k} r_i =n$, there is a partition $T_1, \dots , T_k$ of *P* with $|T_1|=r_1, |T_2|=r_2, \dots , |T_k|=r_k$ such that the centroid of the set of lifted points $T:= T_1 \otimes q_1 \cup \ldots \cup T_k \otimes q_k$ (*this set is also a traversal of* $P_1, \dots , P_n$) has distance less than
  $$\begin{aligned}\delta =\sqrt{\frac{ \varDelta ({\mathcal {G}})}{2(n-1)}}{\text {diam}}(P)\end{aligned}$$
  from the origin ${\mathbf {0}}$.
(ii) The bound is better for the case $n=rk$ and $r_1=\ldots =r_k= n/k$. There exists a partition $T_1, \dots , T_k$ of *P* with $|T_1| = |T_2| = \ldots = |T_k| = r$ such that the centroid of $T:= T_1 \otimes q_1 \cup \ldots \cup T_k \otimes q_k$ has distance less than
  $$\begin{aligned}\gamma = \sqrt{\frac{\Vert {\mathcal {G}}\Vert }{k(n-1)}}{\text {diam}}(P)\end{aligned}$$
  from the origin ${\mathbf {0}}$.

#### Proof

We use an averaging argument to prove the claims, like Adiprasito et al. [1]. More precisely, we bound the average norm $\delta$ of the centroid of the lifted points $T_1 \otimes q_1 \cup \ldots \cup T_k \otimes q_k$ over all partitions of *P* of the form $T_1, \dots , T_k$, for which the sets in the partition have sizes $r_1, \dots , r_k$ respectively, with $\sum _{i=1}^{k} r_i = n$.

#### Proof of Lemma 2.2 (i)

Each such partition can be interpreted as a traversal of the lifted point sets $P_1, \dots , P_n$ that contains $r_i$ points lifted with $q_i$, for $i\in [k]$. Thus, consider any traversal of this type $X=\{x_1,\dots ,x_n\}$ of $P_1,\dots ,P_n$, where $x_a \in P_a$, for $a \in [n]$. The centroid of *X* is $c(X) = (1/n)\sum _{a=1}^{n} x_a$. We bound the expectation $n^2{\mathbb {E}}\bigl (\Vert c(X)\Vert ^2\bigr ) ={\mathbb {E}}\bigl (\bigl \Vert \sum _{a=1}^nx_a\bigr \Vert ^2\bigr )$, over all possible traversals *X*. By the linearity of expectation, ${\mathbb {E}}\bigl (\bigl \Vert \sum _{a=1}^nx_a\bigr \Vert ^2\bigr )$ can be written as

$$\begin{aligned} {\mathbb {E}}\left( \left\| \,\sum _{a = 1}^n x_a\right\| ^2\right)&={\mathbb {E}}\left( \,\sum _{a = 1}^n \Vert x_a\Vert ^2+\sum _{\begin{array}{c} a,b \in [n]\\ a< b \end{array}} \!2\langle x_a,x_b\rangle \right) \\&={\mathbb {E}}\left( \, \sum _{a = 1}^n \Vert x_a\Vert ^2\right) +2{\mathbb {E}}\left( \sum _{\begin{array}{c} a,b \in [n]\\ a < b \end{array}}\!\langle x_a,x_b\rangle \right) . \end{aligned}$$

We next find the coefficient of each term of the form $\Vert x_a\Vert ^2$ and $\langle x_a,x_b\rangle$ in the expectation. Using the multinomial coefficient, the total number of traversals *X* is

$$\begin{aligned} {n \atopwithdelims ()r_1, r_2, \dots , r_k} =\frac{n!}{r_1! r_2!\cdots r_k!}.\end{aligned}$$

Furthermore, for any lifted point $x_a=p_{a,j}$, the number of traversals *X* with $p_{a,j} \in X$ is

$$\begin{aligned}{n - 1 \atopwithdelims ()r_1, \dots , r_j - 1, \dots , r_k}= \frac{(n - 1)!}{r_1! \cdots (r_{j} - 1)! \cdots r_k!}.\end{aligned}$$

So the coefficient of $\Vert p_{a,j}\Vert ^2$ is

$$\begin{aligned}\frac{\displaystyle \frac{(n - 1)!}{r_1! \cdots (r_{j} - 1)! \cdots r_k}}{\displaystyle \frac{n!}{r_1! \cdots r_k!}} = \frac{r_j}{n}.\end{aligned}$$

Similarly, for any pair of points $(x_a,x_b)=(p_{a,i},p_{b,j})$, there are two cases in which they appear in the same traversal: first, if $i=j$, the number of traversals is

$$\begin{aligned}\frac{(n - 2)!}{r_1! \cdots (r_{i} - 2)! \cdots r_k!}.\end{aligned}$$

The coefficient of $\langle p_{a,i},p_{b,j}\rangle$ in the expectation is hence

$$\begin{aligned}\frac{r_i(r_i - 1)}{n(n - 1)}.\end{aligned}$$

Second, if $i\ne j$, the number of traversals is calculated to be

$$\begin{aligned}\frac{(n - 2)!}{r_1! \cdots (r_{i} - 1)! \cdots (r_j - 1)! \cdots r_k!}.\end{aligned}$$

The coefficient of $\langle p_{a,i},p_{b,j}\rangle$ is

$$\begin{aligned}\frac{r_i r_j}{n(n - 1)}.\end{aligned}$$

Substituting the coefficients, we bound the expectation as

$$\begin{aligned}&{\mathbb {E}}\left( \, \sum _{a = 1}^n \Vert x_a\Vert ^2\right) +2{\mathbb {E}}\left( \,\sum _{\begin{array}{c} a,b \in [n]\\ a< b \end{array}} \langle x_a,x_b\rangle \right) = \sum _{a=1}^{n} \sum _{j=1}^{k}\Vert p_{a,j}\Vert ^2 \frac{r_j}{n}\\&\qquad +2\!\sum _{\begin{array}{c} a,b \in [n]\\ a< b \end{array}}\left( \sum _{j = 1}^{k}\langle p_{a,j}, p_{b,j} \rangle \frac{r_j(r_{j} - 1)}{n(n - 1)} +\sum _{\begin{array}{c} i,j \in [k]\\ i \ne j \end{array}} \langle p_{a,i}, p_{b,j} \rangle \frac{r_{i}r_{j}}{n(n-1)}\right) \\&\quad =\sum _{j=1}^{k}\frac{r_j}{n}\sum _{a=1}^{n}\Vert p_{a,j}\Vert ^2\\&\qquad + \frac{2}{n(n -1)}\sum _{\begin{array}{c} a,b \in [n]\\ a< b \end{array}}\left( \,\sum _{i, j \in [k]} \langle p_{a,i}, p_{b,j} \rangle r_{i}r_{j}-\sum _{j=1}^{k} \langle p_{a,j}, p_{b,j} \rangle r_{j}\right) \\&\quad =\sum _{j=1}^{k}r_j \left( \frac{1}{n}\sum _{a=1}^{n}\Vert p_{a,j}\Vert ^2\right) \\&\qquad \qquad +\sum _{\begin{array}{c} a,b \in [n]\\ a< b \end{array}}\,\sum _{i, j \in [k]} \frac{2\langle p_{a,i},p_{b,j}\rangle r_{i}r_{j}}{n(n-1)}-\sum _{\begin{array}{c} a,b \in [n]\\ a < b \end{array}}\,\sum _{j=1}^{k}\frac{2\langle p_{a,j},p_{b,j}\rangle r_{j}}{n(n-1)}. \end{aligned}$$

We bound the value of each of the three terms individually to get an upper bound on the value of the expression. The first term can be bounded as

$$\begin{aligned}&\sum _{j=1}^{k}r_j \left( \frac{1}{n}\sum _{a=1}^{n}\Vert p_{a,j}\Vert ^2\right) =\frac{1}{n}\sum _{j=1}^{k}r_j\left( \,\sum _{a=1}^n\Vert p_{a}\Vert ^2\Vert q_j\Vert ^2\right) \\&\quad =\frac{1}{n}\left( \,\sum _{j=1}^{k}r_j\Vert q_j\Vert ^2\right) \sum _{a=1}^{n}\Vert p_{a}\Vert ^2\le \frac{1}{n}\left( \varDelta ({\mathcal {G}})\sum _{j=1}^{k}r_j\right) \sum _{a=1}^{n}\Vert p_{a}\Vert ^2\\&\quad = \frac{1}{n}(\varDelta ({\mathcal {G}})n)\sum _{a=1}^{n}\Vert p_{a}\Vert ^2<\varDelta ({\mathcal {G}})\frac{n{\text {diam}}(P)^2}{2}, \end{aligned}$$

where we have made use of Lemma 2.1 and the fact that $\sum _{a=1}^{n}\Vert p_{a}\Vert ^2<{n{\text {diam}}(P)^2}/{2}$ (see [1, 4.1]). The second term can be re-written as

$$\begin{aligned}&\sum _{\begin{array}{c} a,b \in [n]\\ a< b \end{array}}\sum _{i, j \in [k]} \frac{2 \langle p_{a,i},p_{b,j}\rangle r_i r_j}{n(n-1)}= \sum _{i, j \in [k]}\frac{2 r_i r_j}{n(n-1)}\sum _{\begin{array}{c} a,b \in [n]\\ a< b \end{array}}\langle p_{a,i},p_{b,j}\rangle \\&\quad =\sum _{i, j \in [k]} \frac{2 r_i r_j}{n(n-1)}\sum _{\begin{array}{c} a,b \in [n]\\ a< b \end{array}}\!\!\langle p_a \otimes q_i, p_b \otimes q_j\rangle \\&\quad =\sum _{i, j \in [k]} \frac{2 r_i r_j}{n(n-1)}\sum _{\begin{array}{c} a,b \in [n]\\ a< b \end{array}}\!\!\langle p_a,p_b\rangle \langle q_i,q_j\rangle \\&\quad =\sum _{i, j \in [k]} \frac{2 \langle q_i,q_j\rangle r_i r_j}{n(n-1)} \sum _{\begin{array}{c} a,b \in [n]\\ a< b \end{array}} \langle p_a,p_b\rangle =\frac{2}{n(n-1)}\sum _{i, j \in [k]}\! \!\langle q_i,q_j\rangle r_i r_j\sum _{\begin{array}{c} a,b \in [n]\\ a < b \end{array}} \!\!\langle p_a,p_b\rangle . \end{aligned}$$

The expression $\sum _{i, j \in [k]} \langle q_i,q_j\rangle r_i r_j$ can be further simplified as

$$\begin{aligned} \sum _{i, j \in [k]} \langle q_i,q_j\rangle r_i r_j&= \sum _{1\le i=j \le k} \langle q_i,q_j\rangle r_i r_j + 2\!\! \sum _{1\le i<j \le k} \langle q_i,q_j\rangle r_i r_j\\&= \sum _{1\le i \le k}\! \Vert q_i\Vert r_i^2 + 2\left( \sum _{v_iv_j\in E} (-1)\cdot r_ir_j \,+\! \sum _{v_iv_j\notin E} \!0\cdot r_ir_j\right) \\&= \sum _{1\le i \le k}\! degree (v_i) r_i^2 \,+\! \sum _{v_iv_j\in E}\!\! -2r_ir_j\\&= \sum _{v_iv_j\in E}\! (r_i^2 + r_j^2 -2r_ir_j)\,=\! \sum _{v_iv_j\in E}\! (r_i-r_j)^2, \end{aligned}$$

where we have again made use of Lemma 2.1. Substituting, the second term becomes

$$\begin{aligned} \frac{2}{n(n-1)}\sum _{(v_i,v_j)\in E}\! (r_i-r_j)^2\sum _{\begin{array}{c} a,b \in [n]\\ a< b \end{array}}\! \langle p_a,p_b\rangle <0, \end{aligned}$$

since we can use $c(P)={\mathbf {0}}$ to bound $\sum _{a, b \in [n], a< b} \langle p_a,p_b\rangle = (-1/2)\sum _{a=1}^{n} \Vert p_a\Vert ^2 <0$. The second term is non-positive and therefore can be removed since the total expectation is always non-negative. The third term is

$$\begin{aligned}&\sum _{\begin{array}{c} a,b \in [n]\\ a< b \end{array}}\,\sum _{j=1}^{k}\frac{-2\langle p_{a,j},p_{b,j}\rangle r_{j}}{n(n-1)}=\sum _{\begin{array}{c} a,b \in [n]\\ a< b \end{array}}\, \sum _{j=1}^{k}\frac{-2\langle p_a\otimes q_j, p_b\otimes q_j\rangle r_{j}}{n(n-1)}\\&\quad =\sum _{\begin{array}{c} a,b \in [n]\\ a< b \end{array}}\,\sum _{j=1}^{k}\frac{-2\langle p_a,p_b\rangle \Vert q_j\Vert ^2 r_j }{n(n-1)}= \sum _{j=1}^{k}\Vert q_j\Vert ^2 r_j\cdot \sum _{\begin{array}{c} a,b \in [n]\\ a< b \end{array}}\frac{-2\langle p_a,p_b\rangle }{n(n-1)}\\&\quad< \sum _{j=1}^{k}\Vert q_j\Vert ^2 r_j\cdot \frac{n{\text {diam}}(P)^2}{2n(n-1)}=\sum _{j=1}^{k}\Vert q_j\Vert ^2 r_j\cdot \frac{{\text {diam}}(P)^2}{2(n-1)}< \frac{n\varDelta ({\mathcal {G}}){\text {diam}}(P)^2}{2(n-1)}. \end{aligned}$$

Collecting the three terms, the expression is upper bounded by

$$\begin{aligned} \frac{{\text {diam}}(P)^2 \varDelta ({\mathcal {G}})n }{2}+ \frac{{\text {diam}}(P)^2\varDelta ({\mathcal {G}})n}{2(n-1)}= & {} \frac{{\text {diam}}(P)^2 \varDelta ({\mathcal {G}})n }{2} \biggl (1+\frac{1}{n-1}\biggr )\\= & {} \frac{{\text {diam}}(P)^2 \varDelta ({\mathcal {G}})n^2 }{2(n-1)} , \end{aligned}$$

which bounds the expectation by

$$\begin{aligned}\frac{1}{n^2}\cdot \frac{{\text {diam}}(P)^2 \varDelta ({\mathcal {G}})n^2 }{2(n-1)}=\frac{{\text {diam}}(P)^2 \varDelta ({\mathcal {G}})}{2(n-1)}. \end{aligned}$$

This shows that there is a traversal such that its centroid has norm less than

$$\begin{aligned} {\text {diam}}(P)\sqrt{\frac{ \varDelta ({\mathcal {G}})}{2(n-1)}}. \end{aligned}$$

$$\square $$

#### Proof of Lemma 2.2 (ii) (Balanced Case)

For the case that *n* is a multiple of *k*, and $r_1=\ldots =r_k={n}/{k}=r$, the upper bound can be improved: the first term in the expectation is

$$\begin{aligned} \sum _{j=1}^{k}r_j \left( \frac{1}{n}\sum _{a=1}^{n}\Vert p_{a,j}\Vert ^2\right)&=\frac{r}{n}\sum _{j=1}^{k} \sum _{a=1}^{n}\Vert p_{a,j}\Vert ^2=\frac{r}{n}\sum _{j=1}^{k} \sum _{a=1}^{n} \Vert p_{a}\Vert ^2 \Vert q_j\Vert ^2\\&=\frac{r}{n}\left( \sum _{j=1}^{k}\Vert q_j\Vert ^2\right) \sum _{a=1}^{n} \Vert p_{a}\Vert ^2=\frac{r}{n}2\Vert {\mathcal {G}}\Vert \sum _{a=1}^{n} \Vert p_{a}\Vert ^2\\&<\frac{r}{n}2\Vert {\mathcal {G}}\Vert \biggl (\frac{n{\text {diam}}(P)^2}{2} \biggr )\le r\Vert {\mathcal {G}}\Vert {\text {diam}}(P)^2, \end{aligned}$$

The second term is zero, and the third term is less than

$$\begin{aligned} \sum _{j=1}^{k}\Vert q_j\Vert ^2 r_j\cdot \frac{{\text {diam}}(P)^2}{2(n-1)}&=r\sum _{j=1}^{k}\Vert q_j\Vert ^2 \cdot \frac{{\text {diam}}(P)^2}{2(n-1)}\\&=2r\Vert {\mathcal {G}}\Vert \frac{{\text {diam}}(P)^2}{2(n-1)}=\frac{r\Vert {\mathcal {G}}\Vert {\text {diam}}(P)^2}{n-1}. \end{aligned}$$

The expectation is upper bounded as

$$\begin{aligned}&n^2{\mathbb {E}}(\Vert c(X)\Vert ^2)< r\Vert {\mathcal {G}}\Vert {\text {diam}}(P)^2+ \frac{r\Vert {\mathcal {G}}\Vert {\text {diam}}(P)^2}{n-1}\\&\implies {\mathbb {E}}(\Vert c(X)\Vert ^2) <\frac{r\Vert {\mathcal {G}}\Vert {\text {diam}}(P)^2 }{n^2}\biggl (1+ \frac{1}{n-1}\biggr )=\frac{r\Vert {\mathcal {G}}\Vert {\text {diam}}(P)^2}{n(n-1)}=\frac{\Vert {\mathcal {G}}\Vert {\text {diam}}(P)^2}{k(n-1)}, \end{aligned}$$

which shows that there is at least one balanced traversal *X* whose centroid has norm less than

$$\begin{aligned}\sqrt{\frac{\Vert {\mathcal {G}}\Vert }{k(n-1)}}{\text {diam}}(P),\end{aligned}$$

as claimed. $$\square $$

## Efficient No-Dimensional Tverberg Theorem

In this section we prove the results of Theorem 1.1:

### Theorem 1.1

(efficient no-dimensional Tverberg)   Let *P* be a set of *n* points in *d* dimensions, and let $k \in \{2, \dots , n\}$ be an integer.

(i) For any choice of positive integers $r_1,\dots ,r_k$ that satisfy $\sum _{i=1}^{k} r_i = n$, there is a partition $T_1, \dots , T_k$ of *P* with $|T_1| = r_1, |T_2|=r_2, \dots , |T_k|=r_k$, and a ball *B* of radius
  $$\begin{aligned} \frac{n{\text {diam}}(P)}{\min _i r_i} \sqrt{\frac{10\lceil {\log _4 k}\rceil }{n-1}}=O\biggl (\frac{\sqrt{n\log k}}{\min _i r_i}{\text {diam}}(P)\biggr ) \end{aligned}$$
  such that *B* intersects the convex hull of each $T_i$.
(ii) The bound is better for the case $n=rk$ and $r_1=\ldots =r_k=r$. There exists a partition $T_1, \dots , T_k$ of *P* with $|T_1| = \ldots = |T_k| = r$ and a *d*-dimensional ball of radius
  $$\begin{aligned} \sqrt{\frac{k(k-1)}{n-1}}{\text {diam}}(P)=O\biggl (\frac{k}{\sqrt{n}}{\text {diam}}(P)\biggr ) \end{aligned}$$
  that intersects the convex hull of each $T_i$.
(iii) In either case, the partition $T_1, \dots , T_k$ can be computed in deterministic time
  $$\begin{aligned} O(nd\lceil {\log k}\rceil ). \end{aligned}$$

### Proof of Theorem 1.1 (i)

We lift the points of *P* to $P_1,\dots ,P_n$ using a graph ${\mathcal {G}}$ and the associated vectors $q_1,\dots ,q_k$ as in Sect. 2.2. The centroid $c(P_a)$ coincides with the origin, for $a \in [n]$. Applying Lemma 2.2, there is a traversal $T:=T_1\otimes q_1\cup \ldots \cup T_k\otimes q_k$ of the lifted points, with $|T_1|=r_1,|T_2|=r_2,\dots ,|T_k|=r_k$, such that its centroid has norm at most $\delta$.

We show that there is a ball of bounded radius that intersects the convex hull of each $T_i$. Let $\alpha _1=r_1/n,\dots ,\alpha _k=r_k/n$ be positive real numbers. The centroid of *T*, *c*(*T*), can be written as

$$\begin{aligned} c(T)&= \frac{1}{n}\sum _{i=1}^{k} \sum _{p\in T_i} p \otimes q_i=\sum _{i=1}^{k} \frac{1}{n}\left( \sum _{p \in T_i} p \right) \otimes q_i\\&=\sum _{i=1}^{k} \frac{r_i}{n}\left( \frac{1}{r_i}\sum _{p \in T_i} p \right) \otimes q_i=\sum _{i=1}^{k} \alpha _i c_i\otimes q_i, \end{aligned}$$

where $c_i = c(T_i)$ denotes the centroid of $T_i$, for $i \in [k]$. Using (1),

2 $$\begin{aligned} \Vert c(T)\Vert ^2&=\left\| \sum _{i=1}^{k} \alpha _i c_i\otimes q_i \right\| ^2=\sum _{v_i v_j\in E} \!\Vert \alpha _i c_i - \alpha _j c_j\Vert ^2. \end{aligned}$$

Let $$x_1=\alpha _1 c_1, x_2=\alpha _2 c_2, \dots , x_k=\alpha _k c_k$$. Then

$$\begin{aligned}\sum _{i=1}^{k} x_i = \sum _{i=1}^{k} \alpha _i c_i =\sum _{i=1}^{k} \frac{r_i}{n}\left( \frac{1}{r_i}\sum _{p\in T_i} p\right) =\frac{1}{n}\sum _{j=1}^{n} p_j = {\mathbf {0}},\end{aligned}$$

so the centroid of $\{ x_1,\dots ,x_k \}$ coincides with the origin. Using $\Vert c(T) \Vert < \delta$ and (2),

$$\begin{aligned}\sum _{v_i v_j\in E} \!\Vert x_i - x_j\Vert ^2=\sum _{v_i v_j\in E} \!\Vert \alpha _i c_i - \alpha _j c_j\Vert ^2<\delta ^2.\end{aligned}$$

We bound the distance from $x_1$ to every other $x_i$. For each $i\in [k]$, we associate to $x_i$ the node $v_i$ in ${\mathcal {G}}$. Let the shortest path from $v_1$ to $v_j$ in ${\mathcal {G}}$ be denoted by $(v_1,v_{i_1},v_{i_2}, \dots , v_{i_z}, v_{j})$. This path has length at most ${\text {diam}}({\mathcal {G}})$. Using the triangle inequality and the Cauchy–Schwarz inequality,

3 $$\begin{aligned} \Vert x_1 - x_j\Vert&\le \Vert x_1 - x_{i_1}\Vert + \Vert x_{i_1} - x_{i_2}\Vert + \dots + \Vert x_{i_z} - x_j\Vert \nonumber \\&\le \sqrt{{\text {diam}}({\mathcal {G}})} \sqrt{\Vert x_1 - x_{i_1}\Vert ^2 +\Vert x_{i_1} - x_{i_2}\Vert ^2 + \dots + \Vert x_{i_z} - x_j\Vert ^2}\nonumber \\&\le \sqrt{{\text {diam}}({\mathcal {G}})}\sqrt{ \sum _{v_iv_j \in E} \Vert x_i - x_j\Vert ^2}< \sqrt{{\text {diam}}({\mathcal {G}})}\,\delta . \end{aligned}$$

Therefore, the ball of radius $\beta :=\sqrt{{\text {diam}}({\mathcal {G}})}\,\delta$ centered at $x_1$ covers the set $\{x_1, \dots , x_k\}$. That means, the ball covers the convex hull of $\{x_1, \dots , x_k\}$ and in particular contains the origin. Using the triangle inequality, the ball of radius $2\beta$ centered at the origin contains $\{x_1, \dots , x_k\}$. Then, the norm of each $x_i$ is at most $2\beta$, which implies that the norm of each $c_i$ is at most $2\beta /\alpha _i$. Therefore, the ball of radius

$$\begin{aligned}\frac{2\beta }{\min _i \alpha _i}= \frac{2n\sqrt{{\text {diam}}({\mathcal {G}})}\,\delta }{\min _i r_i}\end{aligned}$$

centered at ${\mathbf {0}}$ contains the set $\{ c_1,\dots ,c_k \}$. Substituting the value of $\delta$ from Lemma 2.2, the ball of radius

$$\begin{aligned}\frac{2n\sqrt{{\text {diam}}({\mathcal {G}})}}{\min _i r_i}\sqrt{\frac{\varDelta ({\mathcal {G}})}{2(n-1)}}{\text {diam}}(P) =\frac{n{\text {diam}}(P)}{\min _i r_i} \sqrt{\frac{2{\text {diam}}({\mathcal {G}})\varDelta ({\mathcal {G}})}{n-1}}\end{aligned}$$

centered at ${\mathbf {0}}$ covers the set $\{ c_1,\dots ,c_k \}$.

*Optimizing the choice of *${\mathcal {G}}$. The radius of the ball has a term $\sqrt{{\text {diam}}({\mathcal {G}})\varDelta ({\mathcal {G}})}$ that depends on the choice of ${\mathcal {G}}$. For a path graph this term has value $\sqrt{(k-1)2}$. For a star graph, that is, a tree with one root and $k-1$ children, this is $\sqrt{k-1}$. If ${\mathcal {G}}$ is a balanced *s*-ary tree, then the Cauchy–Schwarz inequality in (3) can be modified to replace ${\text {diam}}({\mathcal {G}})$ by the height of the tree. Then, the term is $\sqrt{\lceil \log _s k\rceil (s+1)}$, which is minimized for $s=4$. For this choice of ${\mathcal {G}}$, the radius is bounded by

$$\begin{aligned} \frac{n{\text {diam}}(P)}{\min _i r_i} \sqrt{\frac{10\lceil \log _4 k\rceil }{n-1}},\end{aligned}$$

as claimed.

### Proof of Theorem 1.1 (ii) (Balanced Partition)

For the case $n=rk$ and $r_1=\ldots =r_k=r$, we give a better bound for the radius of the ball containing the centroids $c_1,\dots ,c_k$. In this case, we have $\alpha _1=\alpha _2=\ldots =\alpha _k=r/n=1/k$. Then, (2) is

$$\begin{aligned}\Vert c(T)\Vert ^2=\sum _{v_i v_j\in E} \!\Vert \alpha _i c_i - \alpha _j c_j\Vert ^2=\frac{1}{k^2}\! \sum _{v_i v_j\in E}\! \Vert c_i - c_j\Vert ^2.\end{aligned}$$

Since $\Vert c(T) \Vert < \gamma$, we get

4 $$\begin{aligned} \sum _{v_i v_j\in E}\! \Vert c_i - c_j\Vert ^2 < k^2\gamma ^2. \end{aligned}$$

Similar to the general case, we bound the distance from $c_1$ to any other centroid $c_j$. For each *i*, we associate to $c_i$ the node $v_i$ in ${\mathcal {G}}$. There is a path of length at most ${\text {diam}}({\mathcal {G}})$ from $v_1$ to any other node. Using the Cauchy–Schwarz inequality and substituting the value of $\gamma$ from Lemma 2.2, we get

$$\begin{aligned} \Vert c_1-c_j\Vert&\le \sqrt{{\text {diam}}({\mathcal {G}})}\sqrt{\sum _{v_i v_j\in E} \Vert c_i - c_j\Vert ^2 }<\sqrt{{\text {diam}}({\mathcal {G}})}\,k\gamma \\&=\sqrt{\frac{{\text {diam}}({\mathcal {G}})\Vert {\mathcal {G}}\Vert }{k(n-1)}}k{\text {diam}}(P)=\sqrt{\frac{k}{n-1}}\sqrt{{\text {diam}}({\mathcal {G}})\Vert {\mathcal {G}}\Vert }{\text {diam}}(P). \end{aligned}$$

Therefore, a ball of radius

$$\begin{aligned}\sqrt{\frac{k}{n-1}}\sqrt{{\text {diam}}({\mathcal {G}})\Vert {\mathcal {G}}\Vert }{\text {diam}}(P)\end{aligned}$$

centered at $c_1$ contains the set $c_1,\dots ,c_k$. The factor $\sqrt{{\text {diam}}({\mathcal {G}})\Vert {\mathcal {G}}\Vert }$ is minimized when ${\mathcal {G}}$ is a star graph, which is a tree. We can replace the term ${\text {diam}}({\mathcal {G}})$ by the height of the tree. Then, the ball containing $c_1,\dots ,c_k$ has radius

$$\begin{aligned}\sqrt{\frac{k(k-1)}{n-1}}{\text {diam}}(P),\end{aligned}$$

as claimed.

*As balanced as possible.* When *k* does not divide *n*, but we still want a balanced partition, we take any subset of $n_0=k\lfloor n/k\rfloor$ points of *P* and get a balanced Tverberg partition on the subset. Then, we add the removed points one by one to the sets of the partition, adding at most one point to each set. As shown above, there is a ball of radius less than

$$\begin{aligned}\sqrt{\frac{k(k-1)}{n_0-1}}{\text {diam}}(P)\end{aligned}$$

that intersects the convex hull of each set in the partition. Noting that

$$\begin{aligned}\frac{1}{\sqrt{n_0 - 1}} \le \sqrt{\frac{k+2}{k}}\frac{1}{\sqrt{n-1}},\end{aligned}$$

a ball of radius less than

$$\begin{aligned}\sqrt{\frac{(k+2)(k-1)}{n-1}}{\text {diam}}(P)\end{aligned}$$

intersects the convex hull of each set of the partition.

### Proof of Theorem 1.1 (iii) (Computing the Tverberg Partition)

We now give a deterministic algorithm to compute no-dimensional Tverberg partition $T_1,\dots ,T_k$. The algorithm is based on the method of conditional expectations. First, in Sect. 3.3.1 we give an algorithm for the general case when the sets in the partitions are constrained to have given sizes $r_1,\dots ,r_k$. The choice of ${\mathcal {G}}$ is crucial for the algorithm.

The balanced case of $r_1=\ldots =r_k$ has a better radius bound and uses a different graph ${\mathcal {G}}$. The algorithm for the general case also extends to the balanced case with a small modification, that we discuss in Sect. 3.3.2. We get the same runtime in either case.

#### Algorithm for the General Case

As before, the input is a set of *n* points $P\subset {\mathbb {R}}^d$ and *k* positive integers $r_1,\dots ,r_k$ satisfying $\sum _{i=1}^{k} r_i=n$. Using tensor product construction, each point of *P* is lifted implicitly using the vectors $\{q_1,\dots ,q_k\}$ to get the set $\{P_1,\dots ,P_n\}$. We then compute the required traversal of $\{P_1,\dots ,P_n\}$ using the method of conditional expectations [2], the details of which can be found below. Grouping the points of the traversal according to the lifting vectors used gives us the required partition. We remark that in our algorithm, we do not explicitly lift any vector using the tensor product, thereby avoiding costs associated with working on vectors in $d\Vert {\mathcal {G}}\Vert$ dimensions.

We now describe a procedure to find a traversal that corresponds to a desired partition of *P*. We go over the points in $\{P_1,\dots ,P_n \}$ iteratively in reverse order and find the traversal $Y=(y_1\in P_1,\dots ,y_n\in P_n)$ point by point. More precisely, we determine $y_n$ in the first step, then $y_{n-1}$ in the second step, and so on. In the first step, we go over all points of $P_n$ and select any point $y_n\in P_n$ that satisfies

$$\begin{aligned} {\mathbb {E}}(\Vert c(x_1,x_2,\dots ,x_{n-1},y_n)\Vert ^2)\le {\mathbb {E}}(\Vert c(x_1,x_2,\dots ,x_{n-1},x_n)\Vert ^2). \end{aligned}$$

For the general step, suppose we have already selected the points $\{ y_{s+1},y_{s+2},\dots ,y_n \}$. To determine $y_s$, we choose any point from $P_s$ that achieves

5 $$\begin{aligned} {\mathbb {E}}(\Vert c(x_1,\dots ,x_{s-1},y_{s},y_{s+1},\dots ,y_n)\Vert ^2)\le {\mathbb {E}}(\Vert c(x_1,\dots ,x_{s},y_{s+1},\dots ,y_n)\Vert ^2). \end{aligned}$$

The last step gives the required traversal. We expand the expectation as

$$\begin{aligned}&{\mathbb {E}}(\Vert c(x_1,x_2,\dots ,x_{s-1},y_{s},\dots ,y_n)\Vert ^2)\\&\quad ={\mathbb {E}}\left( \left\| \frac{1}{n}\left( \sum _{i=1}^{s-1}x_i + \sum _{i=s}^{n}y_i \right) \right\| ^2\,\right) =\frac{1}{n^2}{\mathbb {E}}\left( \left\| \left( \sum _{i=1}^{s-1}x_i + \!\sum _{i=s+1}^{n}y_i\right) +y_s\right\| ^2\, \right) \\&\quad =\frac{1}{n^2}\left( {\mathbb {E}}\left( \left\| \sum _{i=1}^{s-1}x_i +\! \sum _{i=s+1}^{n}y_i\right\| ^2 \,\right) + \Vert y_s\Vert ^2 + 2\left\langle y_s, {\mathbb {E}}\left( \sum _{i=1}^{s-1}x_i +\! \sum _{i=s+1}^{n}y_i\right) \right\rangle \right) \\&=\frac{1}{n^2}\left( {\mathbb {E}}\left( \left\| \sum _{i=1}^{s-1}x_i +\! \sum _{i=s+1}^{n}y_i\right\| ^2\,\right) + \Vert y_s\Vert ^2 + 2\left\langle y_s, {\mathbb {E}}\left( \sum _{i=1}^{s-1}x_i\right) + \sum _{i=s+1}^{n}y_i\right\rangle \right) . \end{aligned}$$

We pick a $y_s$ for which ${\mathbb {E}}(\Vert c(x_1,x_2,\dots ,x_{s-1},y_{s},\dots ,y_n)\Vert ^2)$ is at most the average over all choices of $y_s\in P_s$. As the term

$$\begin{aligned} {\mathbb {E}}\left( \left\| \sum _{i=1}^{s-1}x_i + \!\sum _{i=s+1}^{n}y_i\right\| ^2\, \right) \end{aligned}$$

is constant over all choices of $y_s$, and the factor ${1}/{n^2}$ is constant, we can remove them from consideration. We are left with

6 $$\begin{aligned} \begin{aligned}&\Vert y_s\Vert ^2 + 2\left\langle y_s,{\mathbb {E}}\left( \sum _{i=1}^{s-1}x_i\right) + \sum _{i=s+1}^{n}y_i\right\rangle \\&\quad =\Vert y_s\Vert ^2 + 2\left\langle y_s, {\mathbb {E}}\left( \sum _{i=1}^{s-1}x_i\right) \right\rangle + 2\left\langle y_s,\sum _{i=s+1}^{n}y_i\right\rangle . \end{aligned} \end{aligned}$$

Let $y_s = p_s \otimes q_i$ without loss of generality. The first term is

$$\begin{aligned}\Vert y_s\Vert ^2= \Vert p_s \otimes q_i\Vert ^2 = \Vert p_s\Vert ^2\Vert q_i\Vert ^2.\end{aligned}$$

Let $r'_1,\dots ,r'_k$ be the number of elements of $T_1,\dots ,T_k$ that are yet to be determined. In the beginning, $r'_i=r_i$ for each *i*. Using the coefficients from Sect. 2.2, ${\mathbb {E}}\bigl (\sum _{i=1}^{s-1}x_i\bigr )$ can be written as

$$\begin{aligned} {\mathbb {E}}\left( \sum _{i=1}^{s-1}x_i\right)&=\sum _{i=1}^{s-1}\sum _{j=1}^{k}p_{i,j}\frac{r'_j}{s-1}=\sum _{j=1}^{k}\frac{r'_j}{s-1}\sum _{i=1}^{s-1}p_{i,j} =\sum _{j=1}^{k}\frac{r'_j}{s-1}\sum _{i=1}^{s-1} p_i \otimes q_j\\&=\frac{1}{s-1}\sum _{j=1}^{k}r'_j \left( \sum _{i=1}^{s-1} p_i\right) \otimes q_j=\left( \frac{1}{s-1}\sum _{i=1}^{s-1} p_i\right) \otimes \left( \sum _{j=1}^{k}r'_j q_j\right) \\&=c_{s-1} \otimes \left( \sum _{j=1}^{k}r'_j q_j\right) , \end{aligned}$$

where $c_{s-1}={\sum _{i=1}^{s-1} p_i}/({s-1})$ is the centroid of the first $s-1$ points. Using this, the second term can be simplified as

$$\begin{aligned}&2\Bigg \langle y_s,{\mathbb {E}}\Bigg (\sum _{i=1}^{s-1}x_i\Bigg )\Bigg \rangle =2\,\Bigg \langle p_s \otimes q_i, c_{s-1} \otimes \ \Bigg (\sum _{j=1}^{k}r'_j q_j\Bigg ) \Bigg \rangle \\&\quad =2\langle p_s, c_{s-1}\rangle \Bigg \langle q_i,\sum _{j=1}^{k}r'_jq_j\Bigg \rangle =2\langle p_s,c_{s-1}\rangle \Bigg (r'_i\Vert q_i\Vert ^2-\sum _{v_iv_j\in E}\!r'_j\Bigg )=\langle p_s,c_{s-1}\rangle R_i, \end{aligned}$$

where $R_i=2\bigl (r'_i\Vert q_i\Vert ^2-\sum _{v_iv_j\in E} r'_j\bigr )$. The third term is $2\bigl \langle y_s,\sum _{j=s+1}^{n}y_j \bigr \rangle$. Let $y_j=p_j\otimes q_{m_j}$ for $s+1\le j \le n$. The term can be simplified to

$$\begin{aligned} 2\left\langle y_s,\sum _{j=s+1}^{n}y_j \right\rangle&=2\sum _{j=s+1}^{n}\langle y_s,y_j\rangle =2\sum _{j=s+1}^{n}\!\langle p_s \otimes q_i, p_j \otimes q_{m_j}\rangle \\&=2\sum _{j=s+1}^{n} \!\langle p_s,p_j\rangle \langle q_i,q_{m_j}\rangle = 2 \left\langle p_s, \sum _{p\in T_i} p \Vert q_i\Vert ^2 -\!\sum _{j: v_i v_j\in E}\, \sum _{p\in T_j} p\right\rangle \\&={\Bigg \langle {p_s, 2 \Bigg ( \Vert q_i\Vert ^2 \sum _{p\in T_i} p\, -\!\sum _{j: v_i v_j\in E} \,\sum _{p\in T_j} p\Bigg )}\Bigg \rangle }=\langle p_s,U_i\rangle , \end{aligned}$$

where $U_i=2\bigl ( \Vert q_i\Vert ^2 \sum _{p\in T_i} p -\sum _{j: v_i v_j\in E} \sum _{p\in T_j} p\bigr )$ and $T_j$ is the set of points in $p_{s+1},\dots ,p_n$ that was lifted using $q_j$ in the traversal. Collecting the three terms, we get

7 $$\begin{aligned} \Vert p_s\Vert ^2 \Vert q_i\Vert ^2 + \langle p_s,c_{s-1}\rangle R_i + \langle p_s,U_i\rangle =\alpha _s N_i + \beta _s R_i + \langle p_s,U_i\rangle , \end{aligned}$$

with

$$\begin{aligned}N_i=\Vert q_i\Vert ^2,\quad \ \ \alpha _s:=\Vert p_s\Vert ^2,\quad \ \ \beta _s:=\langle p_s,c_{s-1}\rangle .\end{aligned}$$

The terms $\alpha _s,\beta _s,p_s$ are fixed for iteration *s*.

*Algorithm.* For each $s\in [1,n]$, we pre-compute the following:

- prefix sums $\sum _{a=1}^{s} p_{a}$, and
- $$\alpha _s$$ and $$\beta _s$$.

With this information, it is straightforward to compute a traversal in *O*(*ndk*) time by evaluating the expression for each choice of $p_s$. We describe a more careful method that reduces this time to $O(nd\lceil \log k\rceil )$.

We assume that ${\mathcal {G}}$ is a balanced $\mu$-ary tree. Recall that each node $v_i$ of ${\mathcal {G}}$ corresponds to a vector $q_i$. We augment ${\mathcal {G}}$ with the following additional information for each node $v_i$:

- $N_i=\Vert q_i\Vert ^2$: recall that this is the degree of $v_i$.
- $N^{st}_i$: this is the average of the $N_j$ over all elements $v_j$ in the subtree rooted at $v_i$. Since the subtree contains both internal nodes and leaves, this value is not ${\mu +1}$.
- $r'_i$: as before, this is the number of elements of the set $T_i$ of the partition that are yet to be determined. We initialize each $r'_i:=r_i$.
- $R_i=2\bigl (r_i' N_i-\sum _{v_iv_j\in E}r'_j \bigr )$, that is, $r'_i N_i$ minus the $r'_j$ for each node $v_j$ that is a neighbor of $v_i$ in ${\mathcal {G}}$, times two. We initialize $R_i:=0$.
- $R^{st}_i$: this is the average of the $R_j$ values over all nodes $v_j$ in the subtree rooted at $v_i$. We initialize this to 0.
- $T_i, u_i$: as before, $T_i$ is the set of vectors of the traversal that was lifted using $q_i$. The sum of the vectors of $T_i$ is $u_i$. We initialize $T_i=\varnothing$ and $u_i={\mathbf {0}}$.
- $$U_i=2\bigl ( \Vert q_i\Vert ^2 \sum _{p\in T_i} p - \sum _{j: v_i v_j\in E} \sum _{p\in T_j} p \bigr )=2\bigl (u_i N_i-\sum _{v_iv_j\in E} u_j \bigr )$$, initially $${\mathbf {0}}$$.
- $U^{st}_i$: this is the average of the vectors $U_j$ for all nodes $v_j$ in the subtree of $v_i$. $U^{st}$ is initialized as ${\mathbf {0}}$ for each node.

Additionally, each node contains pointers to its children and parents. The quantities $N^{st},R^{st}$ are initialized in one pass over ${\mathcal {G}}$.

In step *s*, we find an $i\in [k]$ for which (7) has a value at most the average

$$\begin{aligned} A_s&=\frac{1}{k}\left( \,\sum _{i=1}^{k} \alpha _s N_i + \beta _s R_i + \langle p_s,U_i\rangle \right) \\&=\frac{\alpha _s}{k} \sum _{i=1}^{k}N_i+ \frac{\beta _s }{k}\sum _{i=1}^{k}R_i+ \left\langle p_s, \frac{1}{k} \sum _{i=1}^{k}U_i \right\rangle =\alpha _s N^{st}_1 + \beta _s R^{st}_1 + \langle p_s,U^{st}_1\rangle , \end{aligned}$$

where $v_1$ is the root of ${\mathcal {G}}$. Then $y_s$ satisfies (5).

To find such a node $v_i$, we start at the root $v_1\in {\mathcal {G}}$. We compute the average $A_s$ and evaluate (7) at $v_1$. If the value is at most $A_s$, we report success, setting $i=1$. If not, then for at least one child $v_m$ of $v_1$, the average for the subtree is less than $A_s$, that is, $\alpha _s N^{st}_m + \beta _s R^{st}_m + \langle p_s,U^{st}_m\rangle < A_s$. We scan the children of $v_1$ and compute the expression to find such a node $v_m$. We recursively repeat the procedure on the subtree rooted at $v_m$, and so on, until we find a suitable node. There is at least one node in the subtree at $v_m$ for which (7) evaluates to less than $A_s$, so the procedure is guaranteed to find such a node.

Let $v_i$ be the chosen node. We update the information stored in the nodes of the tree for the next iteration. We set

- $r'_i:=r'_i-1$ and $R_i:=R_i - 2N_i$. Similarly we update the $R_i$ values for neighbors of $v_i$.
- We set $T_i:=T_i\cup \{p_s\}$, $u_i:=u_i+p_s$, and $U_i:=U_i + 2 N_i p_s$. Similarly we update the $U_i$ values for the neighbors.
- For each child of $v_i$ and each ancestor of $v_i$ on the path to $v_1$, we update $R^{st}$ and $U^{st}$.

After the last step of the algorithm, we get the required partition $T_1,\dots ,T_k$ of *P*. This completes the description of the algorithm.

*Runtime.* Computing the prefix sums and $\alpha _s,\beta _s$ takes *O*(*nd*) time in total. Creating and initializing the tree takes *O*(*k*) time. In step *s*, computing the average $A_s$ and evaluating (7) takes *O*(*d*) time per node. Therefore, computing (7) for the children of a node takes $O(d\mu )$ time, as ${\mathcal {G}}$ is a $\mu$-ary tree. In the worst case, the search for $v_i$ starts at the root and goes to a leaf, exploring $O(\mu \lceil \log _\mu k\rceil )$ nodes in the process and hence takes $O(d\mu \lceil {\log _\mu k}\rceil )$ time. For updating the tree, the information local to $v_i$ and its neighbors can be updated in $O(d\mu )$ time. To update $R^{st}$ and $U^{st}$ we travel on the path to the root, which can be of length $O(\lceil \log _\mu k\rceil )$ in the worst case, and hence takes $O(d\mu \lceil {\log _\mu k}\rceil )$ time. There are *n* steps in the algorithm, each taking $O(d\mu \lceil \log _\mu k\rceil )$ time. Overall, the running time is $O(nd\mu \lceil {\log _\mu k}\rceil )$ which is minimized for a 3-ary tree.

#### Algorithm for the Balanced Case

In the case of balanced traversals, ${\mathcal {G}}$ is chosen to be a star graph as was done in Sect. 3.2. Let $q_1$ correspond to the root of the graph and $q_2,\dots ,q_k$ correspond to the leaves. In this case the objective function $\alpha _s N_i + \beta _s R_i + \langle p_s,U_i\rangle$ from the general case can be simplified:

- for $i=2,\dots ,k$, we have that $R_i=2\bigl (r'_i\Vert q_i\Vert ^2-\sum _{v_iv_j\in E}r'_j\bigr )=2(r'_i-r'_1)$; also, we have
  $$\begin{aligned}U_i=2\left( \sum _{p\in T_i} p \Vert q_i\Vert ^2\, -\!\sum _{ \begin{array}{c} p\in T_j \\ v_i v_j\in E \end{array} }\! p \right) =2\left( \sum _{p\in T_i} p-\sum _{p\in T_1} p \right) ;\end{aligned}$$
- for the root $v_1$, $R_i=2\bigl (r'_i\Vert q_i\Vert ^2-\sum _{v_iv_j\in E}r'_j\bigr )=2\bigl ((k-1) r'_1-\sum _{j=2}^{k}r'_j\bigr )$; also, we can write
  $$\begin{aligned}U_i=2\left( \Vert q_i\Vert ^2 \sum _{p\in T_i} p\, -\!\sum _{ \begin{array}{c} p\in T_j \\ v_i v_j\in E \end{array} }\! p \right) =2\left( (k-1)\sum _{p\in T_i} p - \sum _{p\in \bigcup _{j=2}^{k} T_j}\!\! p \right) .\end{aligned}$$

We augment ${\mathcal {G}}$ with information at the nodes just as in the general case, and use the algorithm to compute the traversal. However, this would need time $O(nd\mu \lceil {\log _\mu k}\rceil )=O(ndk)$ since $\mu =(k-1)$ and the height of the tree is 1. Instead, we use an auxiliary balanced ternary rooted tree ${\mathcal {T}}$ for the algorithm, that contains *k* nodes, each associated to one of the vectors $q_1,\dots ,q_k$ in an arbitrary fashion. We augment the tree with the same information as in the general case, but with one difference: for each node $v_i$, the values of $R_i$ and $U_i$ are updated according to the adjacency in ${\mathcal {G}}$ and not using the edges of ${\mathcal {T}}$. Then we can simply use the algorithm for the general case to get a balanced partition. The modification does not affect the complexity of the algorithm.

## No-Dimensional Colorful Tverberg Theorem

In this section, we prove Theorem 1.2 and give an algorithm to compute a colorful partition.

### Theorem 1.2

(efficient no-dimensional colorful Tverberg)   Let $P_1$, $\ldots$, $P_n\,{\subset }\,\,{\mathbb {R}}^d$ be point sets, each of size *k*, with *k* being a positive integer, so that the total number of points is $N = nk$.

(i) Then, there are *k* pairwise-disjoint colorful sets $A_1,\dots ,A_k$ and a ball of radius
  $$\begin{aligned} \sqrt{\frac{2k(k-1)}{N}}\max _i{\text {diam}}(P_i)= O\biggl (\frac{k}{\sqrt{N}}\max _i{\text {diam}}(P_i)\biggr ) \end{aligned}$$
  that intersects $\mathrm {conv}(A_i)$ for each $i\in [k]$.
(ii) The colorful sets $A_1,\dots ,A_k$ can be computed in deterministic time *O*(*Ndk*).

The general approach is similar to that in Sect. 3, but the lifting and the averaging steps are modified.

### Proof of Theorem 1.2 (i) (Colorful Partition)

Let $q_1,\dots ,q_k$ be the set of vectors derived from a graph ${\mathcal {G}}$ as in Sect. 2. Let $\pi =(1,2,\dots ,k)$ be a permutation of [*k*]. Let $\pi _i$ denote the permutation obtained by cyclically shifting the elements of $\pi$ to the left by $i - 1$ positions. That means,

$$\begin{aligned} \pi _1&= (1,2,\dots ,k-1,k),\\ \pi _2&= (2,3,\dots ,k,1),\\ \pi _3&= (3,4,\dots ,1,2),\\&\dots \\ \pi _{k}&= (k,1,2,\dots ,k-2,k-1). \end{aligned}$$

Let $P_1,\dots ,P_n$ be point sets in ${\mathbb {R}}^d$, each of cardinality *k*. Let $P_1=\{p_{1,1},\dots ,p_{1,k} \}$ and $P_{1,j}=\sum _{i=1}^{k} p_{1,i}\otimes q_{\pi _j(i)}$ be the point in ${\mathbb {R}}^{d\Vert {\mathcal {G}}\Vert }$ that is formed by taking tensor products of the points of $P_1$ with the permutation $\pi _j$ of $q_1,\dots ,q_k$ and adding them up, for $j\in [k]$. For instance, $P_{1,4}=p_1\otimes q_4 + p_2\otimes q_5 + \dots + p_k\otimes q_3$. This gives us a set of *k* points $P'_1=\{P_{1,1},\dots ,P_{1,k} \}$. Furthermore,

8 $$\begin{aligned} \begin{aligned} \sum _{j=1}^{k} P_{1,j}&= \sum _{j=1}^{k}\sum _{i=1}^{k} p_{1,i}\otimes q_{\pi _j(i)}= \sum _{i=1}^{k} \sum _{j=1}^{k} p_{1,i}\otimes q_{\pi _j(i)}\\&=\sum _{i=1}^{k} p_{1,i} \otimes \left( \sum _{j=1}^{k} q_{\pi _j(i)} \right) = \sum _{i=1}^{k} p_{1,i} \otimes \left( \sum _{m=1}^{k} q_{m} \right) ={\mathbf {0}}, \end{aligned} \end{aligned}$$

so the centroid of $P'_1$ coincides with the origin. In a similar manner, for $P_2,\dots ,P_n$, we construct the point sets $P'_2,\dots ,P'_n$, respectively, each of whose centroids coincides with the origin. We now upper bound ${\text {diam}}(P'_1)$. For any point $P_{1,i}$, using (1) we can bound the squared norm as

$$\begin{aligned} \Vert P_{1,i}\Vert ^2&= \left\| \sum _{m=1}^{k} p_{1,m} \otimes q_{\pi _i(m)} \right\| ^2= \left\| \sum _{l=1}^{k} p_{1,\pi _i^{-1}(l)} \otimes q_{l} \right\| ^2\\&=\sum _{v_lv_m\in E} \bigl \Vert p_{1,\pi _i^{-1}(l)} - p_{1,\pi _i^{-1}(m)} \bigr \Vert ^2\le \sum _{v_lv_m\in E}\!\!{\text {diam}}(P_1)^2 \le \Vert {\mathcal {G}}\Vert {\text {diam}}(P_1)^2, \end{aligned}$$

so that $\Vert P_{1,i}\Vert \le \sqrt{\Vert {\mathcal {G}}\Vert }{\text {diam}}(P_1)$. For any two points $P_{1,i},P_{1,j}\in P'_1$,

$$\begin{aligned} \Vert P_{1,i}-P_{1,j}\Vert&\le \Vert P_{1,i}\Vert + \Vert P_{1,j}\Vert \\&\le \sqrt{\Vert {\mathcal {G}}\Vert }{\text {diam}}(P_1) + \sqrt{\Vert {\mathcal {G}}\Vert }{\text {diam}}(P_1)= 2\sqrt{\Vert {\mathcal {G}}\Vert }{\text {diam}}(P_1). \end{aligned}$$

Therefore, ${\text {diam}}(P'_1)\le 2\sqrt{\Vert {\mathcal {G}}\Vert }{\text {diam}}(P_1)$. We get a similar relation for each $P'_{i}$. Now we apply the no-dimensional colorful Carathéodory theorem from [1, Thm. 2.1] on the sets $P'_1,\dots ,P'_n$: there is a traversal $X=\{ x_1\in P'_1,\dots ,x_n\in P'_n \}$ such that

$$\begin{aligned} \Vert c(X)\Vert&< \delta =\frac{\max _{i}{\text {diam}}(P'_i) }{\sqrt{2n}}\\&\le \frac{2\sqrt{\Vert {\mathcal {G}}\Vert }}{\sqrt{2n}} \max _{i}{\text {diam}}(P_i) = \sqrt{\frac{2k\Vert {\mathcal {G}}\Vert }{N}} \max _{i} {\text {diam}}(P_i). \end{aligned}$$

Let $x_1=P_{1,i_1},\dots ,x_n=P_{n,i_n}$ where $1\le i_1,\dots ,i_n\le k$ are the indices of the permutations of $\pi$ that were used. That means,

$$\begin{aligned}x_j=P_{j,i_j}=\sum _{l=1}^{k} p_{j,l}\otimes q_{\pi _{i_j}(l)}=\sum _{m=1}^{k} p_{j,\pi _{i_j}^{-1}(m)}\otimes q_{m}.\end{aligned}$$

Then, we define the colorful sets $A_1,\dots ,A_k$ as

$$\begin{aligned}A_j:=\bigl \{p_{1,\pi _{i_1}^{-1}(i)}, p_{2,\pi _{i_2}^{-1}(i)}, \dots , p_{n,\pi _{i_n}^{-1}(i)} \bigr \},\end{aligned}$$

that is, $A_j$ consists of the points of $P_1,\dots ,P_n$ that were lifted using $q_j$ for $j \in [k]$. By definition, each $A_j$ contains precisely one point from each $P'_i$, so it is a colorful set. Let $c_j$ denote the centroid of $A_j$. We expand the expression

$$\begin{aligned} c(X)&=\frac{1}{n}\sum _{j=1}^{n} P_{j,i_j}= \frac{1}{n}\sum _{j=1}^{n} \sum _{l=1}^{k} p_{j,l} \otimes q_{\pi _{i_j} (l)}= \frac{1}{n}\sum _{j=1}^{n} \sum _{m=1}^{k} p_{j,\pi _{i_j}^{-1} (m)}\otimes q_{m}\\&=\frac{1}{n}\sum _{m=1}^{k} \sum _{j=1}^{n} p_{j,\pi _{i_j}^{-1} (m)}\otimes q_{m}= \frac{1}{n}\sum _{m=1}^{k} \left( \sum _{j=1}^{n} p_{j,\pi _{i_j}^{-1} (m)} \right) \otimes q_{m}\\&= \sum _{m=1}^{k} \frac{1}{n}\left( \sum _{j=1}^{n} p_{j,\pi _{i_j}^{-1} (m)} \right) \otimes q_{m}=\sum _{m=1}^{k} c_m \otimes q_m. \end{aligned}$$

Applying $\Vert c(X)\Vert ^2<\delta ^2$, we get

$$\begin{aligned} \left\| \sum _{m=1}^{k} c_m \otimes q_m \right\| ^2=\sum _{v_l,v_m \in E} \Vert c_l-c_m\Vert ^2 < \delta ^2, \end{aligned}$$

where we made use of (1). Using the Cauchy–Schwarz inequality as in Sect. 3.1, the distance from $c_1$ to any other $c_j$ is at most $\sqrt{{\text {diam}}({\mathcal {G}})}\,\delta$. Substituting the value of $\delta$, this is $\sqrt{{2k{\text {diam}}({\mathcal {G}})\Vert {\mathcal {G}}\Vert }/{N}} \max _{i} {\text {diam}}(P_i)$. Now we set ${\mathcal {G}}$ as a star graph, similar to the balanced case of Sect. 3.2 with $v_1$ as the root. A ball of radius

$$\begin{aligned} \sqrt{\frac{2k(k-1)}{N}} \max _{i}{\text {diam}}(P_i) \end{aligned}$$

centered at $c_1$ contains the set $\{ c_1,\dots ,c_k \}$, intersecting the convex hull of each $A_j$, as required.

### Proof of Theorem 1.2 (ii) (Computing the Colorful Partition)

The algorithm follows a similar approach as in Sect. 3.3. The input consists of the sets of points $P_1,\dots ,P_n$. We use the permutations $\pi _1,\dots ,\pi _k$ of $q_1,\dots ,q_k$ to (implicitly) construct the point sets $P'_1,\dots ,P'_n$. Then we compute a traversal of $P'_1,\dots ,P'_n$ using the method of conditional expectations. This essentially means determining a permutation $\pi _{i_j}$ for each $P'_i$. The permutations directly determine the colorful partition. Once again, we do not explicitly lift any vector using the tensor product, and thereby avoid the associated costs.

We iterate over the points of $\{P'_1,\dots ,P'_n \}$ in reverse order and find a suitable traversal $Y=(y_1\in P'_1,\dots , y_n\in P'_n)$ point by point. Suppose we have already selected the points $\{ y_{s+1},y_{s+2},\dots ,y_n \}$. To find $y_s\in P'_s$, it suffices to choose any point that satisfies

$$\begin{aligned}{\mathbb {E}}(\Vert c(x_1,\dots ,x_{s-1},y_{s},y_{s+1},\dots ,y_n)\Vert ^2)\le {\mathbb {E}}(\Vert c(x_1,\dots ,x_{s},y_{s+1},\dots ,y_n)\Vert ^2).\end{aligned}$$

Specifically, we find the point $y_s$ for which the conditional expectation expressed as ${\mathbb {E}}(\Vert c(x_1,x_2,\dots ,x_{s-1},y_{s},\dots ,y_n)\Vert ^2)$ is minimized. As in (6) from Sect. 3.3, this is equivalent to determining the point that minimizes

9 $$\begin{aligned} \begin{aligned}&\Vert y_s\Vert ^2 + 2\left\langle y_s,{\mathbb {E}}\left( \sum _{i=1}^{s-1}x_i\right) + \sum _{i=s+1}^{n}y_i\right\rangle \\&\quad =\Vert y_s\Vert ^2 + 2\left\langle y_s,{\mathbb {E}}\left( \sum _{i=1}^{s-1}x_i\right) \right\rangle + 2\left\langle y_s,\sum _{i=s+1}^{n}y_i\right\rangle . \end{aligned} \end{aligned}$$

Let $y_s=\sum _{i=1}^{k} p_{s,i} \otimes q_{\pi (i)}$ for some permutation $\pi \in \{\pi _1,\dots ,\pi _k \}$. The terms of (9) can be expanded as

- first term:
  $$\begin{aligned} \Vert y_s\Vert ^2= & {} \left\| \sum _{i=1}^{k} p_{s,i} \otimes q_{\pi (i)} \right\| ^2=\left\| \sum _{l=1}^{k} p_{s,\pi ^{-1}(l)} \otimes q_{l} \right\| ^2\\= & {} \sum _{v_lv_m \in E}\!\Vert p_{s,\pi ^{-1}(l)} -p_{s,\pi ^{-1}(m)}\Vert ^2, \end{aligned}$$
  using (1);
- second term: the expectation can be written as
  $$\begin{aligned}{\mathbb {E}}\left( \sum _{i=1}^{s-1}x_i\right) = \sum _{i=1}^{s-1} \sum _{j=1}^{k} P_{i,j}\frac{1}{k}=\frac{1}{k}\sum _{i=1}^{s-1}\sum _{j=1}^{k} P_{i,j}={\mathbf {0}}, \end{aligned}$$
  as in (8);
- third term: let $\pi _{j_{s+1}},\dots ,\pi _{j_{n}}$ denote the respective permutations selected for $P'_{s+1},\dots ,P'_{n}$ in the traversal. Then
  $$\begin{aligned} \sum _{i=s+1}^{n}y_i&=\sum _{i=s+1}^{n} P_{i,j_{i}}=\sum _{i=s+1}^{n} \sum _{l=1}^{k} p_{i,l}\otimes q_{\pi _{j_{i}}(l)}=\sum _{i=s+1}^{n} \sum _{m=1}^{k} p_{i,\pi ^{-1}_{j_{i}}(m)}\otimes q_{m}\\&=\sum _{m=1}^{k} \left( \sum _{i=s+1}^{n} p_{i,\pi ^{-1}_{j_{i}}(m)} \right) \otimes q_{m}= \sum _{m=1}^{k} \sum _{p\in A'_{m}}p \otimes q_{m}, \end{aligned}$$
  where $A'_m\subseteq A_m$ is the colorful set whose elements from $P_{s+1},\dots ,P_n$ have already been determined. Let $S_m= \sum _{p\in A'_{m}}p$ for each $m=1\dots k$. Then, the third term can be written as
  $$\begin{aligned} 2 \left\langle y_s,\sum _{i=s+1}^{n}y_i \right\rangle&=2\left\langle \sum _{i=1}^{k} p_{s,i} \otimes q_{\pi (i)},\sum _{m=1}^{k} S_{m} \otimes q_{m} \right\rangle \\&= 2 \sum _{i=1}^{k} \sum _{m=1}^{k} \langle p_{s,i} \otimes q_{\pi (i)}, S_{m} \otimes q_{m}\rangle \\&=2 \sum _{l=1}^{k} \sum _{m=1}^{k} \langle p_{s,\pi ^{-1}(l)} \otimes q_{l}, S_{m} \otimes q_{m} \rangle \\&=2 \sum _{l=1}^{k} \sum _{m=1}^{k} \langle p_{s,\pi ^{-1}(l)}, S_{m}\rangle \langle q_{l},q_{m}\rangle \\&=2\sum _{m=1}^{k} \Bigg ( \langle p_{s,\pi ^{-1}(m)}, S_{m}\rangle \Vert q_m\Vert ^2-\!\sum _{v_lv_m\in E}\!\langle p_{s,\pi ^{-1}(l)}, S_{m}\rangle \Bigg )\\&=2\sum _{m=1}^{k} \Bigg \langle \Bigg ( p_{s,\pi ^{-1}(m)}\Vert q_m\Vert ^2 -\! \sum _{v_lv_m\in E}\!p_{s,\pi ^{-1}(l)} \Bigg )\!, S_{m}\Bigg \rangle . \end{aligned}$$

If $\tau$ is the permutation selected in the iteration for $P'_s$, then we update $A'_i=A'_{i}\cup \{p_{s,\tau ^{-1}(i)}\}$ and $S_i=S_i + p_{s,\tau ^{-1}(i)}$ for each $i=1,\dots ,k$.

For each permutation $\pi$, the first and the third terms can be computed in $O(\Vert {\mathcal {G}}\Vert d)=O(kd)$ time. There are *k* permutations for each iteration, so this takes $O(k^2d)$ time per iteration and $O(nk^2d)=O(Ndk)$ time in total for finding the traversal.

#### Remark 4.1

In principle, it is possible to reduce the problem of computing a no-dimensional Tverberg partition to the problem of computing a no-dimensional colorful Tverberg partition. This can be done by arbitrarily coloring the point set into sets of equal size, and then using the algorithm for the colorful version. This can give a better upper bound on the radius of the intersecting ball if the diameters of the colorful sets satisfy

$$\begin{aligned} \max _i {\text {diam}}(P_i)< \frac{{\text {diam}}(P_1\cup P_2\cup \ldots \cup P_n)}{\sqrt{2}}. \end{aligned}$$

However, the algorithm for the colorful version has a worse runtime since it does not utilize the optimizations used in the regular version.

## No-Dimensional Generalized Ham-Sandwich Theorem

We prove Theorem 1.3 in this section:

### Theorem 1.3

(no-dimensional generalized Ham-Sandwich)   Let *k* finite point sets $P_1$, $\ldots$, $P_k$ in ${\mathbb {R}}^d$ be given, and let $m_1, \dots , m_k$, $2 \le m_i \le |P_i|$ for $i \in [k]$, $k\le d$, be any set of integers.

(i) There is a linear transformation and a ball $B\in {\mathbb {R}}^{d-k+1}$ of radius
  $$\begin{aligned} (2 + 2\sqrt{2}) \max _i \frac{{\text {diam}}(P_i)}{\sqrt{m_i}}, \end{aligned}$$
  such that the hypercylinder $B\times {\mathbb {R}}^{k-1}\subset {\mathbb {R}}^d$ has depth at least $\lceil |P_i|/m_i\rceil$ with respect to $P_i$, for $i \in [k]$, after applying the transformation.
(ii) The ball and the transformation can be determined in time
  $$\begin{aligned} O\left( d^6+dk^2+\sum _{i} |P_i|d\right) . \end{aligned}$$

This is a no-dimensional version of a generalization of the Ham-Sandwich theorem [33]. We briefly describe the history of the problem before detailing the proof.

The centerpoint theorem was proven by Rado in [26]. It states that for any set of *n* points $P\subset {\mathbb {R}}^d$, there exists some point $\mathrm {cp}(P)\in {\mathbb {R}}^d$, called the *centerpoint* of *P*, such that $\mathrm {cp}(P)$ has depth at least $\lceil n/(d+1)\rceil$. The centerpoint generalizes the concept of median to higher dimensions. The theorem can be proven using Helly’s theorem [17] or Tverberg theorem.

The Ham-Sandwich theorem [33] shows that for any set of *d* finite point sets $P_1,\dots ,P_d\subset {\mathbb {R}}^d$, there is a hyperplane *H* which bisects each point set, that is, each closed halfspace defined by *H* contains at least $\lceil |P_i|/2\rceil$ points of $P_i$, for $i \in [d]$. The result follows by an application of the Borsuk–Ulam theorem [18].

Živaljević and Vrećica [37] and Dol’nikov [13], independently, proved a generalization of these two results for affine subspaces (*flats*):

### Theorem 5.1

Let $P_1,\dots ,P_k$ be $k\le d$ finite point sets in ${\mathbb {R}}^d$. Then there is a $(k-1)$-dimensional flat *F* of depth at least $|P_i|/(d-k+2)$ with respect to $P_i$, for $i \in [k]$.

For $k=1$, this corresponds to the centerpoint theorem while for $k=d$, this is the Ham-Sandwich theorem, and thereby interpolates between the two extremes.

We prove a no-dimensional version of this theorem, where $1/(d-k+2)$ can be relaxed to be an arbitrary but reasonable fraction. In fact, we prove a slightly stronger version that allows an independent choice of fraction for each point set $P_i$ individually. The idea is motivated by the result of Bárány et al., who showed in [6] that under certain conditions of “well-separation”, *d* compact sets $S_1,\dots ,S_d\subset {\mathbb {R}}^d$ can be divided by a hyperplane that such the positive half-space contains an $(\alpha _1,\dots ,\alpha _d)$-fraction of the volumes of $S_1,\dots ,S_d$, respectively. A discrete version of this result for finite point sets was proven by Steiger and Zhao in [32], which they term as the *generalized Ham-Sandwich theorem*. Our result can be interpreted as a no-dimensional version of this result, but we do not have constraints on the point sets as in [6, 32].

Without loss of generality, we assume that the centroid $c(P_1)={\mathbf {0}}$. We first approach a simpler case:

### Lemma 5.1

Let $c(P_1)=\ldots =c(P_k)={\mathbf {0}}$ and $m_1, \dots , m_k$, $2 \le m_i \le |P_i|$ for $i \in [k]$, be any choice of integers. Then the ball of radius

$$\begin{aligned}(2+2\sqrt{2})\max _i \frac{{\text {diam}}(P_i)}{\sqrt{m_i}}\end{aligned}$$

centered at ${\mathbf {0}}$ has depth at least $\lceil |P_i|/m_i\rceil$ with respect to $P_i$, for $i \in [k]$.

### Proof

Consider any point set $P_i$ and a no-dimensional $\lceil {|P_i|}/{m_i}\rceil$-partition of $P_i$. From [1, Thm. 2.5], we know that the ball *B* centered at $c(P_i)={\mathbf {0}}$ of radius

$$\begin{aligned}(2+\sqrt{2}){\text {diam}}(P_i)\sqrt{\frac{\lceil |P_i|/m_i\rceil }{|P_i|}}<(2+\sqrt{2}){\text {diam}}(P_i) \sqrt{\frac{2}{m_i}}=\frac{(2+2\sqrt{2}){\text {diam}}(P_i)}{\sqrt{m_i}}\end{aligned}$$

intersects each set of the partition. Let *H* be any half-space that contains *B*. We claim that *H* contains at least one point from each set in the partition. Assume for contradiction that *H* does not contain any point from a given set in the partition. Then, the convex hull of that set does not intersect *H*, and hence *B*, which is a contradiction. This shows that *B* has depth $\lceil |P_i|/m_i\rceil$. Let $B'$ be the ball of radius $(2+2\sqrt{2})\max _i{\text {diam}}(P_i)/\sqrt{m_i}$ centered at the origin. Then $B'$ has depth at least $\lceil |P_i|/m_i\rceil$ with respect to $P_i$ for each $i=1,\dots ,k$. $\square$

We prove an auxiliary result that will be helpful in proving the main result:

### Lemma 5.2

Let $P_1,\dots ,P_k \subset {\mathbb {R}}^{d_1}$ be finite point sets. Let *v* be any vector in ${\mathbb {R}}^{d_1}$ and project $P_1,\dots ,P_k$ on the hyperplane *H* via ${\mathbf {0}}$ with normal *v*. If some set $X\subset H$ has depth $\alpha _1,\dots ,\alpha _d$ respectively for the projected point sets, then $X\times {\mathbb {R}}_v\subset {\mathbb {R}}^{d_1}$ has the same depths for the original point sets, where ${\mathbb {R}}_v$ is the one dimensional subspace containing *v*.

### Proof

Consider any half-space ${\mathcal {H}}\subset {\mathbb {R}}^{d_1}$ that contains $X\times {\mathbb {R}}_v$. Then ${\mathcal {H}}$ contains ${\mathbb {R}}_v$, so it can be written as $\hat{{\mathcal {H}}}\times {\mathbb {R}}_v$, where $\hat{{\mathcal {H}}}\subset H$ is a half-space containing *X*. $\hat{{\mathcal {H}}}$ contains at least $\alpha _i$ points of each $P_i$. By orthogonality of the projection, ${\mathcal {H}}$ also contains at least $\alpha _i$ points of each $P_i$, proving the claim. $\square$

### Proof of Theorem 1.3 (i)

Given point sets $P_1,\dots ,P_k$ with $c(P_1)={\mathbf {0}}$, we apply orthogonal projections on the points multiple times so that their centroids coincide. In the first step, we set $v_1=c(P_2)$. Let $l_1$ be the line through the origin containing $v_1$ and let $H_{v_1}$ be the hyperplane via ${\mathbf {0}}$ with normal $v_1$. Let $f_1:{\mathbb {R}}^d \rightarrow H_{v_1}$ be the orthogonal projection defined as $f(p)=p-\langle p,v\rangle {v}/{|v|^2}$. Let $P_1^{1},\dots ,P_k^{1}\subset {\mathbb {R}}^{d-1}$ be the point sets obtained by applying the orthogonal projection on $P_1,\dots ,P_{k}$, respectively. Under this projection $c(P_1^{1})=c(P_2^{1})={\mathbf {0}}$. In the next step we set $v_2=c(P_3^{1})$ and define $l_2$ and $H_{v_2}$ analogously. We project $P_1^{1},\dots ,P_k^{1}$ onto $H_{v_2}$ to get $P_1^{2},\dots ,P_k^{2}$ with $c(P_1^{2})=c(P_2^{2})=c(P_3^{2})={\mathbf {0}}$. We repeat this process $k-1$ times to get a set of points $P_1^{k-1},\dots ,P_k^{k-1}\subset {\mathbb {R}}^{d-k+1}$ with $c(P_1^{k-1})=\ldots =c(P_k^{k-1})={\mathbf {0}}$. Using Lemma 5.1, there is a ball *B* of radius

$$\begin{aligned}(2+2\sqrt{2})\max _i \frac{{\text {diam}}(P_i^{k-1})}{\sqrt{m_i}}<(2+2\sqrt{2})\max _i\frac{{\text {diam}}(P_i)}{\sqrt{m_i}}\end{aligned}$$

of the required depth. Applying Lemma 5.2 on $P_1^{k-2},\dots ,P_k^{k-2}\subset {\mathbb {R}}^{d-k+2}$, $B\times \ell _{k-1}$ also has the required depth. Repeated application of Lemma 5.2 gives us $B\times \ell _{k-1}\times \ell _{k-2}\times \dots \times \ell _{1}$. Since the Cartesian product may have more than *d* co-ordinates, we apply a linear transformation so that the subspace spanned by the orthogonal set $\ell _1,\dots ,\ell _{k-1}$ is ${\mathbb {R}}^{k-1}$. Then, $B\times {\mathbb {R}}^{k-1}$ has the desired properties. $\square$

### Proof of Theorem 1.3 (ii)

To compute the vectors $v_1,\dots ,v_{k-1}$, we note that

$$\begin{aligned}v_i=c(P_{i+1}^{i-1})=c(f_{i-1}\circ f_{i-2}\circ \dots \circ f_{1}(P_{i+1}^{i-1}))=f_{i-1}\circ f_{i-2}\circ \dots \circ f_{1} (c(P_{i+1}^{i-1})),\end{aligned}$$

by linearity of the projection. Therefore, at the beginning we first compute each centroid $c(P_i)$ and in each step we apply the projection on the relevant centroids. The projection is applied $1+\dots +k-2=O(k^2)$ times. Computing the centroid in the first step takes $O\bigl (\sum _{i}|P_i| d\bigr )$ time. Computing the projection once takes *O*(*d*) time, so in total $O(dk^2)$ time. Finding the linear transformation takes another $O(d^6)$ time. $\square$

## Conclusion and Future Work

We gave efficient algorithms for a no-dimensional version of Tverberg theorem and for a colorful counterpart. To achieve this end, we presented a refinement of Sarkaria’s tensor product construction by defining vectors using a graph. The choice of the graph was different for the general- and the balanced-partition cases and also influenced the time complexity of the algorithms. It would be interesting to find more applications of this refined tensor product method. Another option could be to look at non-geometric generalizations based on similar ideas. It would also be interesting to consider no-dimensional variants other generalizations of Tverberg’s theorem, e.g., in the tolerant setting [22, 30].

The radius bound that we obtain for the Tverberg partition is $\sqrt{k}$ off the optimal bound in [1]. This seems to be a limitation in handling (4). It is not clear if this is an artifact of using tensor product constructions. It would be interesting to explore if this factor can be brought down without compromising on the algorithmic complexity. In the general partition case, setting $r_1=\ldots =r_k$ gives a bound that is $\sqrt{\lceil {\log k}\rceil }$ worse than the balanced case, so there is some scope for optimization. In the colorful case, the radius bound is again $\sqrt{k}$ off the optimal [1], but with a silver lining. The bound is proportional to $\max _i{{\text {diam}}(P_i)}$ in contrast to ${\text {diam}}{(P_1\cup \ldots \cup P_n)}$ in [1], which is better when the colors are well separated.

The algorithm for colorful Tverberg theorem has a worse runtime than the regular case. The challenge in improving the runtime lies a bit with selecting an optimal graph as well as the nature of the problem itself. Each iteration in the algorithm looks at each of the permutations $\pi _1,\dots ,\pi _k$ and computes the respective expectations. The two non-zero terms in the expectation are both computed using the chosen permutation. The permutation that minimizes the first term can be determined quickly if ${\mathcal {G}}$ is chosen as a path graph. This worsens the radius bound by $\sqrt{k-1}$. Further, computing the other (third) term of the expectation still requires *O*(*k*) updates per permutation and therefore $O(k^2)$ updates per iteration, thereby eliminating the utility of using an auxiliary tree to determine the best permutation quickly. The optimal approach for this problem is unclear at the moment.

## Data Availability

Data sharing not applicable to this article as no datasets were generated or analyzed during the current study.

## Footnotes

1 We note that this identity is very similar to the Laplacian quadratic form that is used in spectral graph theory; see, e.g., the lecture notes by Spielman [31] for more information.
